# 2025 Japanese Heart Rhythm Society / Japanese Circulation Society Consensus Statement on the Appropriate Use of Ambulatory and Wearable Electrocardiographs

**DOI:** 10.1002/joa3.70059

**Published:** 2025-05-23

**Authors:** Takanori Ikeda, Takashi Ashihara, Yu‐ki Iwasaki, Maki Ono, Nobuyuki Kagiyama, Takehiro Kimura, Kengo Kusano, Ritsuko Kohno, Keita Saku, Tetsuo Sasano, Keitaro Senoo, Seiji Takatsuki, Naohiko Takahashi, Mitsuru Takami, Yukiko Nakano, Kenichi Hashimoto, Katsuhito Fujiu, Tadashi Fujino, Atsushi Mizuno, Koichiro Yoshioka, Eiichi Watanabe, Wataru Shimizu, Koichi Node

**Keywords:** Ambulatory electrocardiogram (ECG) devices, Implantable loop recorders, Oscillometric blood pressure measurement, Photoplethysmography, Wearable electrocardiogram (ECG) devices

## Abstract

Recently, some clinicians have been diagnosing and treating arrhythmias on the basis of electrocardiogram (ECG) devices with low accuracy. In Europe and the US, several statements on the use of ECGs have already been published by related academic societies. In addition, with the relaxation of regulations on media advertising ambulatory/wearable ECG devices, the frequency of use of simple ECG devices by the general public will increase in the future. Therefore, this statement describes the functions and features of non‐invasive ambulatory or wearable ECG devices that have been approved as medical devices in Japan (and that can record ECGs remotely), as well as points to note when using them; provides an overview of data storage and security for ambulatory/wearable ECG devices and implantable loop recorders (ILRs), as well as discussing differences between their use and the use of non‐invasive ambulatory/wearable ECG devices; and provides classes of recommendation for the use of these devices and their evaluation for each arrhythmia type or condition. We describe lead‐based ambulatory ECG devices (classical 24‐h Holter ECG monitoring), handheld ECG devices, handheld‐based ECG devices using a smartphone, wearable ECG devices (smartwatch and garment ECG devices), and patch ECG devices. In addition, we provide information on methods that are not based on the original ECG, such as photoplethysmography and oscillometric blood pressure measurement, and describe the limitations of their use. We hope that the publication of this statement will lead to the appropriate use of ambulatory/wearable ECG devices in Japan.

## 1 INTRODUCTION

This statement describes the functions and features of non‐invasive ambulatory or wearable electrocardiograph (ECG) devices that have been approved as medical devices in Japan (and that can record ECGs remotely), as well as points to note when using them. The background to the publication of this statement is that there are some clinicians who are diagnosing and treating arrhythmias using ECGs with low accuracy. In Europe and the US, several statements on the use of ECGs have already been published by related academic societies.^1^, ^2^, ^3^, ^4^ In addition, with the relaxation of regulations on media advertising for ambulatory/wearable ECG devices, the frequency of use of simple ECGs will increase not only among medical professionals but also among the general public.

ECG devices include lead‐based ambulatory ECG devices (classic 24‐h Holter monitoring), patch ECG devices, handheld ECG devices, handheld‐based ECG devices using smartphones, and smartwatch ECG devices. Many of these are marketed as “ambulatory ECG devices”, but those that are worn, such as smartwatch ECG devices and garment ECG devices, are called “wearable ECG devices”. The patch ECG devices, which have become widely used in recent years, are positioned between ambulatory and wearable ECG devices. This statement describes the basics of these types of ECG devices and their appropriate use, and includes information on recording methods that are not based on the original ECG, such as photoplethysmography (PPG) and oscillometric blood pressure measurement, as well as limitations regarding their use. In addition, we provide an overview of data storage and security for ambulatory/wearable ECG devices. We also refer to implantable loop recorders (ILRs), and discuss differences between their use and the use of non‐invasive ambulatory/wearable ECG devices. Finally, we provide classes of recommendation for the use of these devices and their evaluation for different arrhythmia types or conditions.

We hope that the publication of this statement will lead to the appropriate use of ambulatory/portable ECG devices in Japan. In this context, a green heart symbol in the consensus statement table means “recommended for use”, a yellow heart means “may be used”, and a red heart means “should not be used”.

## 2 TYPES AND CHARACTERISTICS OF AMBULATORY / PORTABLE ECG DEVICES

### 2.1 Lead‐Based Ambulatory ECG Devices

Up until now, lead‐based ambulatory ECG devices that can record ECGs for long periods of time have been used for the diagnosis of arrhythmia and ischemic heart disease. This is what is known as the classic or standard Holter ECG. It is based on 2 or 3 leads, but it is also possible to record 12 leads using multiple electrodes. It is also possible to display a 12‐lead ECG recording using XYZ signals derived from lead vectors. Recording 12 leads makes it possible to calculate the QT interval, QT/RR, and QT dispersion, detect Brugada‐type waveforms, determine the origin of ventricular arrhythmias, and estimate the site of ischemia. However, it should be noted that the quality of ECGs recorded with the trunk placement of limb electrodes is not the same as that of standard 12‐lead ECGs, because the R‐wave amplitude changes due to the QRS axis shifting to the right.^5^

With some models, depending on the frequency characteristics, it is possible to perform additional analysis of late potentials (LPs) and repolarization instability indicators (T wave alternans [TWA], T wave variability) from microvolt recordings using a high sampling rate (1000 Hz). In long‐term recordings of ≥4 h, the detection rate of paroxysmal arrhythmia increases, and it becomes possible to observe daily variance by displaying the histogram of the duration of atrial fibrillation (AF burden) and measuring heart rate variability (HRV; the cyclic variation in heart rate). Conversely, continuous wearing of devices is a burden for patients, so it is recommended that recordings be made for ≤48 h. Some models are equipped with a 3‐axis accelerometer, which can record body position (supine, right lateral, left lateral, prone, sitting, standing) and exercise intensity (metabolic equivalents), and can be used to diagnose exertional symptoms, asymptomatic arrhythmias, and cardiac neurosis.

For pacemaker pulses and noise, the detection channel can be selected before recording starts, and the display can be enlarged on a tablet or personal computer (PC). The latest Holter monitoring devices are lightweight (with the smallest model weighing just 13 g) and waterproof, making them more convenient for patients. Electrodes include disposable electrodes with integrated lead wires that have a 2‐layer shield structure that is resistant to static electricity noise, and electrodes with an integrated main unit that can be worn inside clothing.

Among the ambulatory ECG devices with leads, external loop ECG devices have an automatic trigger recording function. The possibility to set automatic recognition protocols for bradycardia, tachycardia, extrasystoles, and unstable RR intervals also makes it possible to detect asymptomatic arrhythmias. The Monitoring of Syncopes and/or Sustained Palpitations of Suspected Arrhythmic Origin (SYNARR‐FLASH) study enrolled 395 patients with syncope or palpitations suspected of being caused by arrhythmia, and the diagnostic rate over a 4‐week period was 24.5% for syncope and 71.6% for palpitations.^6^ In response to this, the European guidelines recommended the use of an ambulatory loop recorder as a diagnostic tool to be used prior to an implantable loop recorder (ILR) as a step‐by‐step diagnostic tool.^6^ Conversely, ambulatory loop recorders lack the ability to identify the origin of arrhythmia due to the lack of spatial vector information for P, QRS, T waves and the ST segment, and they do not have the ability to continuously record heart rhythm. During the recording period, it is necessary to continuously attach electrodes to the patient, and the analysis results depend on patient compliance.

Table **1** shows ambulatory ECG devices with leads and external loop ECG devices that can be used in Japan at present. There are 9 ambulatory ECG device models with leads from 5 companies.

**TABLE 1:** Types and Characteristics of Lead‐Based Ambulatory Electrocardiogram Devices Used in Japan

	Lead‐based ambulatory ECG devices (2‐channel / 3‐channel / 12‐lead)				
Manufacturer	FUKUDA DENSHI		NIHON KOHDEN		
Product name	FM‐1400	FM‐1500	RAC‐2512	RAC‐5103	RAC‐5203
Appearance	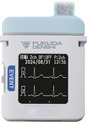	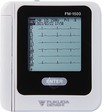	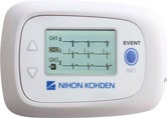	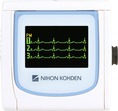	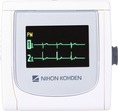
Size (W × ×H × D; mm)	40 × 41 × 10 (excluding protrusions)	60 × 65 × 13 (excluding protrusions)	81.3 × 57.8 × 19.5	53.8 × 53.8 × 17.1	53.8 × 53.8 × 17.8
Weight (g)	17 (with battery case, excluding ECG connector, electrodes, and cable)	49 (excluding batteries and card)	50 (excluding batteries and card)	41.5 (excluding batteries and card)	43.5 (excluding batteries and card)
Waterproof	Yes	Yes	–	Yes	Yes
Recording channels	2‐channel, 3‐channel	2‐channel, 3‐channel, 12‐lead	2‐channel, 3‐channel, 12‐lead, 3‐channel bipolar, (X, Y, Z)	2‐channel, 3‐channel	2‐channel, 3‐channel, 3‐channel bipolar, (X, Y, Z)
Recording time	24 h	Up to 7 days	Up to 3 days	24 h	Up to 7 days
High sampling rate (Hz)	–	1000	1000	–	1000
Other features		• LP, TWA measurement • QT, QT/RR measurement • Brugada waveform measurement	• Estimation of PVC origin • Capture of ST changes		• LP measurement possible

	Lead‐based ambulatory ECG devices (2‐channel / 3‐channel / 12‐lead)			External loop ECG device	
Manufacturer	KENZMEDICO		GE HealthCare	MicroPort® CRM	
Product name	Cardy 305 pico	Cardy 1201	SEER 1000	SpiderView®	SpiderFlash™
Appearance	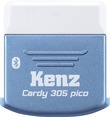	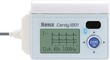	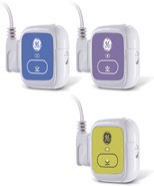	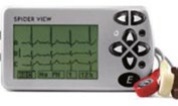	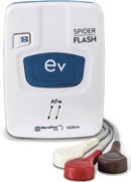
Size (W × H × D; mm)	35.8 × 40.8 × 8.7	61.6 × 46.3 × 21.6	71.0 × 64.0 × 20.0	97 × 54 × 23	53 × 74 × 19.5
Weight (g)	13	45 (excluding batteries)	53	85	50
Waterproof	Yes	Yes	–	–	–
Recording channels	3‐channel (CM5, NASA, CC5/auxiliary lead)	1‐channel, 2−/3‐channel, 12‐lead	2−/3‐channel	2−/3‐channel, XYZ calculation, 12‐lead	2‐channel
Recording time	Up to 2 days	Up to 2 days (12‐lead) Up to 7 days (2−/3‐channel) Up to 10 days (1‐channel)	24 h/48 h/7 days	Up to 4 days	Up to 40 days
High sampling rate (Hz)	–	1000	–	1000	–
Other features	• Body position and exercise intensity recorded using 3‐axis acceleration sensor • Detailed symptoms, behavior and time recorded using attached event recorder	• QT measurement • PVC origin estimation • Capture of ST changes • Enhanced pacemaker pulse detection (2‐channel) • Simple event recording possible using the main unit event button; alternatively, detailed symptoms, behavior, and time can be recorded using the event recorder	• Connects to Bluetooth‐compatible PCs and tablets • Can record for up to 7 days on a single AAA 1.5‐V alkaline battery	• LP, TWV measurement • QT, QT/RR measurement • Pacemaker spike detection	• 24 h on the first day, event loop ECG from the second day onwards • Auto trigger or patient activation can be set • Fixed time recording: up to 20 locations/day • Records all RR intervals • Can be removed during bathing and reattached after bathing

ECG, electrocardiogram; LP, late potential; PC, personal computer; PVC, premature ventricular contraction; TWA, T wave alternans; TWV, T wave variability.

### 2.2 Patch ECG Devices and Garment ECG Devices

Patch ECG devices and garment ECG devices are medical devices that continuously record the ECG waveform during a patient's daily life over a long period of time. Patch and garment ECG devices are approved as a simple Holter ECG, and are positioned somewhere between ambulatory and wearable ECG devices. The main use of these devices is to detect irregular heartbeats that are difficult to detect with conventional ECGs that can record for short periods of time or up to 24 h (normal 12‐lead ECGs or lead‐based ambulatory ECG devices). The design of these small, lightweight patch ECG devices is generally such that they are attached directly to the chest. This means that there is no need to use lead wires, reducing the burden on the patient when wearing the device. Many of these devices are also waterproof, so they can be used even when bathing. In cases where long‐term ECG recording is required, there are devices that can be used continuously for up to 14 days. In devices with event buttons, the patient presses the button when symptoms occur to mark the episode.^7^ Clinically important arrhythmias with symptoms are often detected in the first week of long‐term ECG measurement.^3^

Some of the patch and garment ECG devices are attached at medical institutions and returned to the hospital, but in many cases the device is sent directly from a company to a patient's home, under instruction from a doctor based on a contract between the doctor and the company. After the test, the patient mails the device back to the company. This new system is expected to improve the rate of implementation of the test for examinees who are unable to or have difficulty attending medical institutions due to busy schedules, old age, and geographical conditions. As such, patch and garment ECG devices can be used by a wide range of examinee groups. The latest ECG devices perform real‐time analysis of recorded ECGs, enabling faster data collection and diagnosis.^2^

The patch and garment ECG devices that can record for long periods of time are particularly effective for detecting paroxysmal atrial fibrillation (AF) and asymptomatic arrhythmia. They are useful for capturing the occurrence of arrhythmia when it is accidental and short‐lived. They are particularly effective for detecting AF, and have high accuracy rates and diagnostic results. The patch and garment ECG devices are particularly effective for screening for AF when the frequency of AF is only around ≤15% per day. The results following the use of a 2‐week ambulatory ECG device in a population with moderate to high risk of AF are comparable to those obtained using an IRL for 2 weeks, and the detection rate of AF with 2‐week ambulatory ECG devices is 10‐fold higher than that of standard treatment using auscultation and palpation alone.^8^, ^9^, ^10^

Table **2** lists patch and garment ECG devices currently available in Japan. There are several models on the market with different features that should be considered given the environment and purpose of use of the device. The features of each of the devices are described below. Note that ECG data analysis is performed using each company's proprietary software, but there is also an artificial intelligence (AI) cloud‐based program (the long‐term ECG analysis SmartRobin AI series) that is compatible with multiple models and specializes in the diagnosis of AF from long‐term ECG recordings.

**TABLE 2:** Comparison of Patch and Garment Electrocardiogram Devices for Long‐Term Recording

Manufacturer	FUKUDA DENSHI	JSR	KENZMEDICO	Japan Lifeline	
Product name	eMemo (WR‐100)	Heartnote®	Simple Holter®	AT‐Patch (ATP‐C70)	AT‐Patch (ATP‐C130)
Appearance	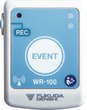	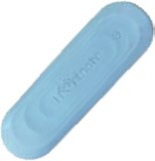	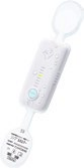	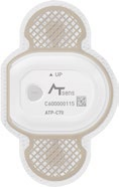	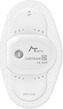
Release date	May 2017	August 2020	October 2022	October 2022	Certified: Not yet on sale
Manufacture and sales	FUKUDA DENSHI	JSR	KENZMEDICO	Japan Lifeline	
Manufacturer	FUKUDA DENSHI	JSR	Seki Aoi Techno	Atsens (Republic of Korea)	
Size (W × H × D; mm)	33 × 44 × 13 (excluding protruding parts)	30 × 100 × 5	30 × 66 × 6.7 (excluding electrodes) 31 × 164 × 7.5 (including electrodes)	39.6 × 32.6 × 7.7 (excluding electrodes) 47.1 × 74 × 8.4 (including electrodes)	39 × 31 × 7.8 (excluding electrodes) 48.8 × 84 × 8.5 (including electrodes)
Weight (g)	25 (including battery)	12 (including battery)	15 (including battery and electrodes)	13	
Reuse of main unit	○	× (charged and maintained by retailer)	×	×	
Battery	Lithium primary battery ×1 (CR2450)	Lithium polymer rechargeable battery ×1 (built‐in)	Lithium primary battery ×1 (built‐in CR2032)	Lithium primary battery ×1 (built‐in CR2032)	
Recording time	Up to 14 days	Up to 7 days	Up to 24 h	Up to 7 days	Up to 14 days
Recording medium	Internal flash memory	Internal flash memory	Internal flash memory	Internal flash memory	
No. channels	Bipolar 1‐channel	Bipolar 1‐channel	Bipolar 1‐channel	Bipolar 1‐channel	
Sampling frequency (Hz)	125	256	125	250	
Body position/movement	×	○ 3‐axis accelerometer	○ 3‐axis accelerometer	○ 3‐axis accelerometer	
Event button	○	×	○	○	
Waterproof	IPX6/IPX8	IPX4/IPX7	IP66/IP68	IP44	IP57
Electrode	External	Integrated (using adhesive or dressing film)	Integrated	Integrated	
Waveform confirmation method	Viewer software (Bluetooth)	–	LED level meter	Viewer software/viewer app (Bluetooth)	
Analysis	Analysis device/analysis software	1. Outsourced (mailed) 2. AI analysis (AI analysis is specialized for AF)	Outsourced (mailed)	AI analysis software (AT‐report)	
Saving as PDF at hospital	Possible	Possible	Possible	Possible	
Manufacturer data storage period	Saved at medical institution	0.5 years	14 days (back‐up period up to 2 years)	Saved at medical institution	
Service life (years)	6	3	2	1	
Expiry date	–	1 month after shipping	2 years after manufacture	1 year after manufacture	
Other features			• Single use per device per examination • Analysis reports are delivered via a dedicated website • Analysis reports include the results of arrhythmia interpretation by specialist physicians		

Manufacturer	durantis	Philips	Toyota Tsusho	kokoromil	Toray Medical
Product name	gram®	ePatch®	LOTUS HEART®	eclat®	hitoe™
Appearance	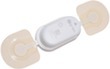	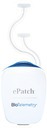	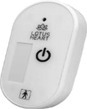	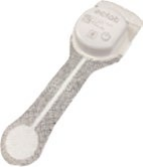	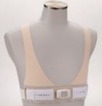
Release date	January 2023	July 2023	April 2024	August 2024	January 2018
Manufacture and sales	durantis	Philips Japan	HAMADA	kokoromil	ECG machine: Parama‐Tech Wearable electrodes: Toray Medical
Manufacturer	Miyako Marantz	Braemar Manufacturing, LLC (USA)	HAMADA	Miyako Marantz	–
Size (W × H × D; mm)	163 × 33 × 14.5	40 × 49 × 12	63 × 33 × 14.5	45 × 50 × 14	ECG machine: 58.5 × 17.5 × 38.5 Wearable electrodes: small, medium, large
Weight (g)	~19 (including battery)	20	~25 (including battery)	~15 (including battery)	~31
Reuse of main unit	×	○	○	×	○
Battery	Primary battery ×1 (built‐in)	Battery (built‐in, USB rechargeable)	Lithium primary battery ×1 (CR2450)	Lithium primary battery ×1 (built‐in)	Lithium polymer rechargeable battery ×1 (built‐in)
Recording time	Up to 7 days	Up to 10 days	Up to 7 days	Up to 7 days	Up to 14 days
Recording medium	–	–	Internal flash memory	Built‐in memory	Internal flash memory
No. channels	Bipolar 1‐channel	Patch electrode: 5 days, bipolar 2‐channel 10 days, bipolar 1‐channel	Bipolar 1‐channel	Bipolar 1‐channel	1‐channel (CC5)
Sampling frequency (Hz)	250	256	250	250	125
Body position/movement	–	–	–	–	○ 3‐axis accelerometer
Event button	×	○ Double‐tap the center of the main unit	Not on the main unit, but can be input using a paired smartphone	–	○
Waterproof	IP57	IPX4	IPX4	○	IPX2
Electrodes	Built‐in	External (patch type)	External	Built‐in	External (dedicated wearable electrodes)
Waveform confirmation method	–	–	Viewer app (Bluetooth connection)	–	Viewer software
Analysis	Outsourced (cloud)	AI analysis software (Cardiologs Platform)	Outsourced (cloud)	Outsourced (cloud)	1. Outsourced 2. AI analysis
Saving as PDF at hospital	Possible	Possible	Possible	Possible	Possible
Manufacturer data storage period	5 years	Permanent	5 years	5 years	0.5 years (2 years in case of original data before analysis)
Service life	–	300 charge/discharge cycles or 2 years	3 years	–	5 years (ECG)
Expiry date	2 years	2 years	–	2 years	Displayed on the packaging (wearable electrodes)
Other features	• Single use per test per device	• Test kit sent from Philips at the request of the medical institution and can be put on and worn by the patient themselves • AF detection accuracy of 98.5% with AI analysis • HRV, QTc, and CVHR can be measured	• Waveforms can be checked on a smartphone • Recorded data are managed in the cloud in real time • Analysis can begin immediately after the test is finished • The electrode attachment/detachment system reduces the burden on the patient	• Single use for one test per device	• Can be put on and taken off like normal clothing • Wearable electrodes can be washed at home

AF, atrial fibrillation; AI, artificial intelligence; AT, atrial tachycardia; CVHR, cyclic variation of heart rate; HRV, heart rate variability; LED, light‐emitting diode; QTc, correct QT interval.

#### 2.2.1 eMemo WR‐100, Made by FUKUDA DENSHI

The eMemo WR‐100 has a longest recording time of up to 14 days. The eMemo WR‐100 cannot record body position or movement using an accelerometer. But it does have an event button. It is possible to check the waveform from a tablet device after attaching the eMemo WR‐100 to the tablet. The device can be reused, and medical institutions need to purchase the main unit.

#### 2.2.2 Heartnote®, Made by JSR

The Heartnote® can record for up to 7 days. Medical institutions do not need to purchase the device. The Heartnote® is thin, lightweight, flexible, and cordless, so it is easy for patients to wear. The Heartnote® is waterproof, so patients can shower or take a bath while wearing it, and although it does not have an event button, it does have an acceleration sensor. In addition to normal arrhythmia analysis, it is also possible to use the AI analysis service (SmartRobin AI cloud service) provided by Cardio Intelligence, which specializes in AF.

#### 2.2.3 Simple Holter®, Made by KENZMEDICO

The Simple Holter® is a patch ECG device that has an acceleration sensor and event button. It is a single‐use device, and can record for up to 24 h per test. The Simple Holter® is waterproof, so patients can bathe while wearing it. The light‐emitting diode (LED) level meter allows the wave height to be checked while the device is being worn, and the position of the device can be adjusted. The Simple Holter® has a shelf‐life of 2 years from the date of manufacture, and can be stocked for this period.

#### 2.2.4 AT‐Patch, Made by Japan Lifeline

The maximum recording time for the AT‐patch is 7 days. The AT‐patch is a disposable type of device, with both an acceleration sensor and an event button, and there is no need for an initial investment. It is possible to check the waveform via a Bluetooth connection while the device is being worn. In addition, once recording has started, the power cannot be turned off, so there is no risk of accidentally turning it off while in use.

#### 2.2.5 ePatch®, Made by Philips

The ePatch® can record for up to 5 days with 1 electrode patch, and replacing the patch allows recording for up to 10 days. The ePatch® can be mailed to a patient's home, so there is no need for medical institutions to purchase the device. The electrodes are adhesive, and it is possible to record on 2 bipolar channels (for 10 days: 1 bipolar channel if 2 patches are used). The ePatch® has an event button, but lacks an accelerometer. In addition to detecting AF, the ePatch® can measure HRV and QT time. Although patients cannot take a bath while wearing the device, they can take a shower.

#### 2.2.6 gram®, Made by Durantis, and eclat®, Made by Kokoromil

With these devices, measurements can be made for up to 7 days. These devices are single‐use devices, and medical institutions can purchase the number of devices they need and use them whenever they want (valid for 2 years). The devices are attached to the body using only electrode pads, so there is little burden on the patient. The devices are waterproof, so can be used when showering, but not when taking a bath. The gram® comes with an analysis service provided by kokoromil using the medical device program duranta Analysis® made by durantis, whereas the eclat® comes with an analysis service provided by kokoromil using the medical device program kokorotoku® made by kokoromil. Both products are sold by kokoromil.

#### 2.2.7 LOTUS HEART®, Made by Toyota Tsusho

The LOTUS HEART® can record for up to 7 days. The main unit can be reused, in which case commercially available electrodes are used. The LOTUS HEART® is used in conjunction with a smartphone, with data transferred to the smartphone via Bluetooth and then sent to a cloud server. There is no event button on the main unit, but it is possible to check the ECG waveform and enter events via the app on the smartphone.

#### 2.2.8 hitoe™, Made by Toray Medical

The hitoe™ can record for up to 14 days. It uses a special garment with built‐in dry electrodes that do not require adhesive that is worn over the patient's clothes in combination with an ECG. The patient can wear and remove the garment as they would with normal clothing while measurements are being taken, and the garment can be washed at home after the ECG is removed. The hitoe™ can also be sent to a patient's home, so there is no need for medical institutions to purchase the device.

### 2.3 Handheld ECG Devices

#### 2.3.1 Conventional Handheld ECG Devices, in Which the Main Unit Is Pressed Against the Chest With the Hand

Handheld ECG devices are easy to carry around and, because the patient records the ECG by pressing the device against their chest or hand as needed, they are non‐invasive and cause little stress on the patient. The general consumer name for these devices is “cardiac activity recorder for ictal events.”^11^ Previously, the terms “ambulatory event electrocardiograph” or “non‐loop event recorder” were often used,^12^, ^13^ but more recently, in order to clarify the differences from other types of ECGs, it has become more common to refer to them as “handheld ECG devices.” Handheld ECG devices first appeared around 2005, and although there have been various transitions, they are currently sold by several companies. There are various types of handheld ECG devices, including those that can be used alone, those that can be used in conjunction with smartphones (see next section), those that can record multiple leads simultaneously, those that can transmit data to a PC or cloud for analysis, and those that have analysis support functions. OMRON's HCG‐801® and HCG‐901® are commonly used as stand‐alone devices, but there are also many other devices on the market.

Handheld ECG devices are mainly used when the patient is aware of symptoms, such as arrhythmia or ischemic heart disease. They are particularly useful when the frequency of occurrence is not high, around a few times per month. In a meta‐analysis comparing handheld ECG devices and 24‐h Holter monitoring in screening for AF, the 19 min of recording using handheld ECG devices was comparable to that of Holter monitoring.^14^ Handheld ECG devices can also be used to prove conditions such as cardiac neurosis, which are symptomatic but not cardiovascular diseases.^7^ Even in the absence of symptoms, handheld ECG devices can be useful for detecting asymptomatic arrhythmias if the timing of the recording is roughly specified, such as recording twice a day, in the morning and in the evening.

Conversely, although the accuracy of ECG recordings is high and they can be easily repeated, handheld ECG devices are limited to about 30 s per recording, and, except for special devices, they are usually limited to 1 channel of induction, they cannot record in situations such as when the subject is asleep or bathing, the patients' own ECGs are recorded by themselves and not by other people, they cannot be used in patients with implanted cardiac devices, and they cannot be used in conjunction with magnetic resonance imaging. Thus, there are limitations as to the clinical situations in which handheld ECG devices are useful.

In Japan, as a medical fee, 150 points can be claimed per medical examination in “3 Electrocardiogram examination using ambulatory electrocardiogram recording and transmission device for ictal events” under “D208 Electrocardiogram Examination,” regardless of the number of ECGs recorded. However, this claim is allowed only when an ambulatory ECG recording and transmission device for ictal events is used to record an ECG in outpatients. It should be noted that if the device has not been certified by the Ministry of Health, Labor and Welfare of Japan as a medical device that can be covered by insurance (a special maintenance and management medical device; this applies to the Apple Watch®), costs cannot be reimbursed.

#### 2.3.2 Handheld ECG Devices Using a Smartphone

**FIGURE 1:**
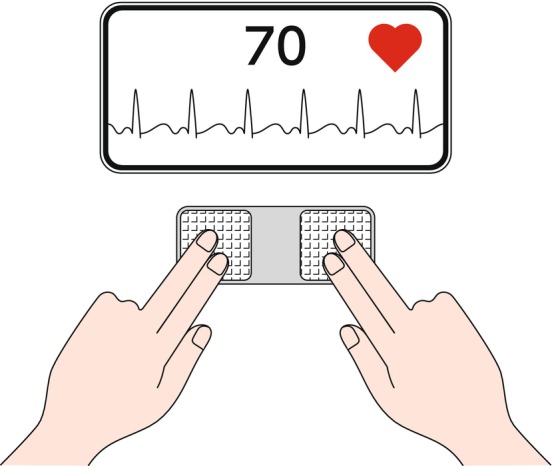
Recording an electrocardiogram (ECG) using a handheld‐based ECG device connected to a smartphone.

OMRON's HCG‐8060 T® (AliveCor's KardiaMobile® 6 L in Europe and the US) and HCG‐8010 T1® are handheld ECGs devices that use a smartphone to record and store ECGs. To use these devices, the dedicated app (OMRON connect) needs to be installed on the patient's smartphone in advance. There are 2 ways to record an ECG with the HCG‐8060 T® one is to place both hands on the ends of the device and record only 1 lead (Figure **1**), and the other is to place the thumb on the electrode on the surface of the main unit, attach the back of the unit to the left foot, and calculate a pseudo‐6‐lead ECG waveform. The ECG waveform is displayed on a smartphone via Bluetooth, and the results of the automatic analysis are displayed after a minimum recording of 30s. The waveform can be output as a PDF file, and can be printed or sent by email. In terms of medical fees, the use of these devices can be claimed as “3 Electrocardiogram examination using ambulatory electrocardiogram recording and transmission device for ictal events” under “D208 Electrocardiogram Examination”, with points to be noted are described in Section 3.1 above.

In Europe and the US, the KardiaMobile® 6 L has been used for some time. Previous studies have evaluated its accuracy in detecting AF and QTc intervals.^15^, ^16^, ^17^, ^18^, ^19^, ^20^ Compared with the results of 12‐lead ECGs read by specialist physicians, the automated analysis of the KardiaMobile® 6 L had a sensitivity of 96.6%, a specificity of 94.1%, and a kappa coefficient of 0.89 for detecting AF.^15^ In addition, when the 6‐lead ECG waveforms of the KardiaMobile® were compared with those of other smartwatch‐type ECG devices, cardiologists found that the KardiaMobile® was superior in detecting AF.^17^ Furthermore, when the rate of AF detection over a 1‐year period was compared between screening for AF using the device's twice‐weekly ECG test and regular outpatient follow‐up in patients aged ≥65 years, the KardiaMobile® was shown to be approximately 4‐fold more effective than regular outpatient follow‐up.^21^

Table **3** lists the types of conventional and smartphone‐based handheld ECG devices currently available in Japan.

**TABLE 3:** Types of Ambulatory Handheld Electrocardiogram Devices (as of August 2024)

Manufacturer	OMRON HEALTHCARE					Parama‐Tech
Product name	OMRON Portable ECG HCG‐801®	OMRON Portable ECG HCG‐901®	OMRON Portable ECG HCG‐8060 T®	OMRON Portable ECG HCG‐8010 T1®	OMRON Portable ECG HCG‐9010 U®	Portable ECG EP‐501
Appearance	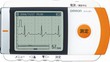	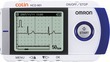	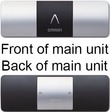	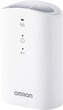	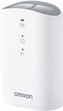	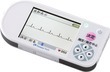
General name	Cardiac activity recorder during paroxysmal AF					Cardiac activity recorder during paroxysmal AF
Main unit weight (g)	~130 (including batteries)	~140 (including batteries)	~24 (including batteries)	~130 (including batteries)		~90
External dimensions (mm)	121 × 67 × 24		30 × 90 × 7.4	83 × 53 × 30		120 × 15 × 58
Power source	DC 3 V (2 AAA alkaline batteries)		DC 3 V (1 CR2016 lithium battery)	DC 3 V (2 AAA alkaline batteries)		DC 3.7 V (lithium rechargeable) DC 5 V (dedicated AC)
Lead method and no. channels	Bipolar 1 (V4 equivalent recommended)	Bipolar 1 (V4 equivalent recommended), with external electrodes	1 lead: bipolar 1 6 lead: bipolar 3/unipolar 3	Bipolar 1		Bipolar 1 (V4 equivalent/I recommended), with external electrodes
Recording time	30 s	30 s SD card: 30, 60, 90, 120, 150, or 180 s	30 s, 1, 2, 3, 4, or 5 min	30 s		30, 60, 90, 120, 150, 180, 210, 240, 270, or 300 s, or continuous
Communication method	–	USB	Bluetooth LE		USB 2.0	Wi‐Fi/USB
Display	Graphic LCD, monochrome		Use smartphone dedicated app		ECG management/interpretation support software	Color LCD 2.8 inch
No. records	5 (internal memory) 300 (SD memory card)	15 (internal memory) 300 (SD memory card)	Storage limit in smartphone app	10 (internal memory) Storage limit in smartphone app	100 (internal memory)	500 (*μ*SD memory card)
Battery life	~400 recordings		~12 months of use	~400 recordings		~500 recordings
Rhythm analysis	Heart rate (fast/slow), irregular heartbeat, irregular waveform, no irregularities, analysis not possible	Presence/absence of subjective symptoms, event detected/not‐detected, analysis not possible (noise/recording interruption)	Normal sinus rhythm, bradycardia, tachycardia, possibility of AF, unclassifiable, analysis not possible	Heart rate (fast/slow), irregular pulse, irregular waveform, no irregularity, unable to analyze	ECG management software: Heart rate (fast/slow), irregular pulse, irregular waveform, no irregularity, unable to analyze Interpretation support software: Displays detailed information on waveform changes	Normal, heart rate (fast/slow), irregular heart rate, irregular waveform
Measurement items other than rhythm	Heart rate					Heart rate, RR trend
Support software	ECG printing software HCG‐SOFT‐2 (Windows)	Interpretation support software HCG‐SOFT‐CL1 (Windows)	Smartphone app OMRON connect (iOS/Android)		ECG management software: HCG‐SOFT‐3 (Windows) Interpretation support software: HCG‐SOFT‐CL2 (Windows)	Dedicated viewer software
Notes	• Sampling 125 Hz • Heart rate measurement range 2–200 beats/min	• Sampling 125 Hz • Heart rate measurement range 40–200 beats/min	• Developed by AliveCor, Inc. • Sampling 300 Hz • Heart rate measurement range 30–300 beats/min	• Sampling 125 Hz • Heart rate measurement range 30–200 beats/min		• Sampling: 250 Hz • MFER format adopted

Manufacturer	SAN‐EI MEDISYS				SIMPLEX QUANTUM	LifeWatch Technologies
Product name	Health Monitor Checkme Pro X	Health Monitor Checkme Pro B ADV	Health Monitor Checkme Lite ADV	Health Monitor Checkme ECG ADV	Shinden‐kun	Card Guard CG‐2100 Event Recorder
Appearance	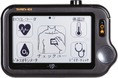	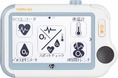	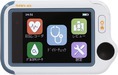	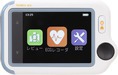	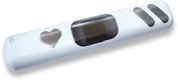	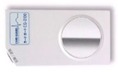
General name	Cardiac activity recorder during paroxysmal AF/pulse oximeter/ infrared skin thermometer		Cardiac activity recorder during paroxysmal AF/pulse oximeter	Cardiac activity recorder during paroxysmal AF	Cardiac activity recorder during paroxysmal AF	Transmission‐type ECG
Main unit weight (g)	~68		~60		~120	~55
External dimensions (mm)	88 × 56 × 13				198 × 30 × 39	105 × 55 × 8
Power source	DC 3.7 V, 560 mAh (rechargeable lithium ion battery, AC 100–240 V, 50/60 Hz, 2 Wh)				DC 3 V (2 AA alkaline batteries)	DC 3 V (2 CR2032 lithium batteries)
Lead method and no. channels	Bipolar 1 (I/II recommended) No. electrodes on back: 2	Bipolar 1 (I/II recommended) No. electrodes on back: 1			Bipolar 1 (I/II recommended)	Bipolar 1
Recording time	30, 60, or 90 s	30, 60, 90, or 300 s, or 8 h (lead cord)	30, 60, or 90 s		30 s	32 s
Communication method	Bluetooth LE/USB cables				LTE	Transmission and reception with the ECG automatic analysis center
Display	Monochrome touch screen 2.7 inch		Color touch screen 2.4 inch		Color LCD 1.28 inch	
No. records	100 (for 30 s)				No limit	1
Battery life	–				~120 recordings	~1500 h (~2000 recordings)
Rhythm analysis	Normal, tachycardia, bradycardia, irregular, impossible to analyze				Normal, irregular heartbeat (tachycardia, bradycardia, irregular)	
Measurement items other than rhythm	Heart rate, QRS width, QT/QTc, ST level, SpO_2_ (continuous for 8 h with an ECG oxygen adapter), body temperature, no. steps		Heart rate, QRS width, ST level (lead code), SpO_2_	Heart rate, QRS width, ST level (lead code)	Heart rate, LF/HF (aLF/aHF ratio)	Heart rate, RR, PR, QRS width, ST level, QT/QTc
Support software	Checkme Viewer (Windows/iOS/Android/Cloud)				Shinden‐kun (iOS/Android/Web)	
Notes	• Sampling 500 Hz • Transcutaneous arterial blood oxygen saturation can be measured at 35 points; 100 points overnight • Can be recorded by someone other than the patient (Checkyou)	• Sampling 500 Hz • Can claim 35 points for measurement of transcutaneous arterial blood oxygen saturation; 100 points for overnight measurement	• Sampling 250 Hz • Can claim 35 points for measurement of transcutaneous arterial blood oxygen saturation	• Sampling 250 Hz	• Sampling 600 Hz • Biological data analysis AI cloud AssistMedica research support cloud service available	• Communication method: via smartphone app • Display: Heart Care ECG service

All devices in Table 3 are controlled medical devices requiring special maintenance and can be claimed for 150 points; the unit price per point is 10 yen. aHF, absolute power of high frequency; aLF, absolute power of low frequency; ECG, electrocardiogram; HF, high frequency; LCD, liquid crystal display; LE, low enagy; LF, low frequency; MFER, Medical waveform Format Encoding Rules.

### 2.4 ECG Recording

Using a SmartwatchIn recent years, wearable ECG devices have become more widespread, with ECG recording using a smartwatch being a prime example. In addition, there are various other types of wearable ECG devices, such as necklace, bracelet, glasses, ring, and T‐shirt devices. For patients who have only a few episodes of irregular heartbeat, it is not possible to carry an ambulatory ECG device around all the time, but smartwatches can be worn as part of a person's regular attire and have the advantage of not missing any recording opportunities. Conversely, the effects of noise resulting from a poor fit of the device and body movement are a common disadvantage of wearable ECG devices. It is important to remember that the ECG recorded by a smartwatch can only be used as an aid in the diagnosis of arrhythmia, and that arrhythmia should not be diagnosed on the basis of these recordings alone. As such, the accuracy of the ECG recorded using a smartwatch is not equivalent to that of a normal ECG. The reason for this is that smartwatches display ECGs using PPG (see below).

**FIGURE 2:**
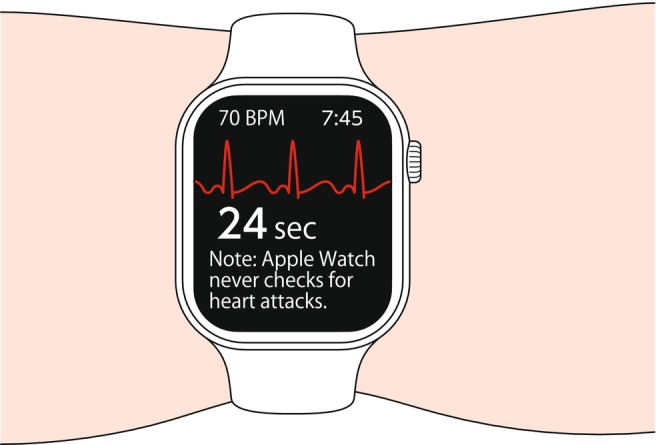
Recording an electrocardiogram (ECG) using an ECG app from Apple Inc.

At present, the only home‐use medical device program available in Japan is the Apple Watch® ECG app (Figure **2**). The Apple Watch® is a smartwatch made by Apple that works in conjunction with an iPhone, and it is equipped with a home ECG program that allows individuals to actively record their own ECG with their own arm.^22^, ^23^ When a person touches the crown of their head while seated, a waveform similar to that of Lead I of an ECG is recorded, and the results are displayed in 5 categories: sinus rhythm, AF, high heart rate, low heart rate, and undetermined. With regard to arrhythmia, only AF with a specific range of pulse rates is detected. For many users, recording an ECG will be a new experience, and they should be aware that the results displayed may not necessarily be correct, because noise often affects the analysis. The recorded ECG can be output as a PDF, so it is possible to have it read by a specialist at a later date. In addition, because symptoms can also be registered at the time of recording, the output can be used to clarify causal relationships between the displayed results and symptoms.

### 2.5 ECG‐Equipped Blood Pressure Monitors

**FIGURE 3:**
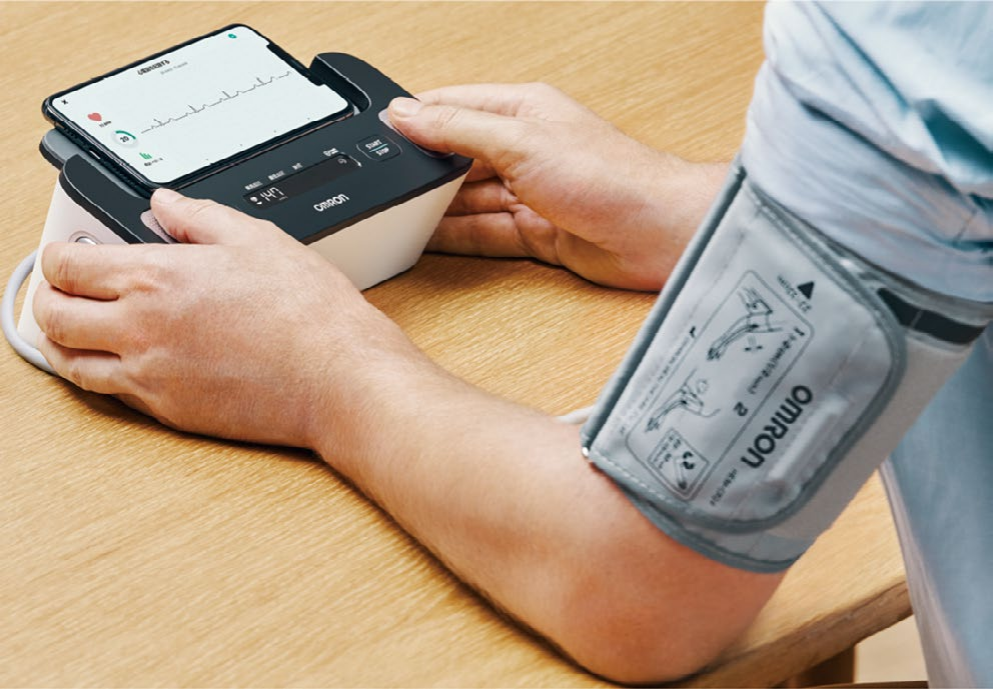
Simultaneous recording of blood pressure and an electrocardiogram (ECG) using an ECG‐equipped blood pressure monitor. (Image courtesy of OMRON HEALTHCARE Co., Ltd.)

The HCR‐7800 T® series of upper arm blood pressure monitors with ECG manufactured by OMRON fall into the category of ECG‐equipped blood pressure monitors (Figure **3**). Generic names for this type of product include automatic electronic blood pressure monitor, ambulatory ECG recording and transmission device during attacks, and program for ambulatory ECG recording and transmission device during attacks, and the medical device class classification is a controlled medical device. Each recording is 30 s long and finishes while the blood pressure is being measured. The results of the recording are divided into 6 categories: normal sinus rhythm, bradycardia, tachycardia, possible AF, cannot classify, and cannot analyze. The accuracy of these devices for detecting AF is high, with a sensitivity of 96–98% and a specificity of 86–96%.^24^, ^25^, ^26^

In terms of medical fees, the use of these devices can be claimed as “3 Electrocardiogram examination using ambulatory electrocardiogram recording and transmission device for ictal events” under “D208 Electrocardiogram Examination”, with points to be noted as described in Section 3.1 above. Therefore, when a patient visits their doctor, they will need to install the corresponding smartphone app OMRON connect (because the ECG is recorded on the app) and share the information with the doctor. In addition, if the doctor confirms AF in the recorded ECG waveform, they can make a definitive diagnosis of AF.

ECG‐equipped blood pressure monitors are useful not only for detecting new cases of AF,^25^ but also for monitoring recurrence after AF treatment.^26^ By increasing the number of opportunities to detect recurrence by using an ECG device that can be used at home rather than conventional monitoring methods (such as 12‐lead ECG or Holter monitoring), the likelihood of detecting recurrence early and starting appropriate treatment increases. The main causes of false‐positive results with these home‐use ECGs are often ECG baseline drift and premature atrial beats.^24^

### 2.6 Heart Rate Monitors Using PPG

Photoplethysmography (PPG) uses LEDs and light detectors that are mounted on smartwatches and other various ambulatory and wearable ECG devices to measure changes in blood volume and obtain information about the pulse wave associated with the heartbeat. Because PPG can monitor pulse patterns in real time and detect changes in heart rate, it enables early screening for irregular heartbeats. In a meta‐analysis (evaluating 7623 cases) of the use of PPG to detect AF, the pooled estimates of the sensitivity and specificity of PPG were 94.7% and 97.6%, respectively.^27^ There are currently various smartwatches on the market using PPG, and research studies have been published on the use of the Apple Watch® and Huawei Watch®, both with in‐built PPG, to detect AF^28^, ^29^ (see Chapter II, Section 6 and Chapter V, Section 1).

In Japan, the iAide®2 portable device with built‐in PPG has obtained regulatory certification. This device has a function that allows the checking of continuous pulse rate data converted from detected pulse waves online for 24 h, but it does not have a program for detecting arrhythmia. Conversely, the Apple Watch® has the 2 programs described below in Sections 6.1 and 6.2, which have been approved as home heart rate monitoring programs. Because the Apple Watch® itself is not a medical device, users and medical professionals need to be aware of the limitations of its functions when using it.

#### 2.6.1 Irregular Heartbeat Notification Program

The irregular heartbeat notification program is a home heart rate monitoring program that determines whether the fluctuations in pulse rate collected by the Apple Watch® are likely to be AF.^30^ The clinical usefulness of this notification was evaluated in the Apple Heart Study, a clinical study in the US, with the results showing that the notification led to the diagnosis of asymptomatic or undiagnosed AF.^28^ The function of the application is to determine whether or not the fluctuation in pulse rate is likely to be AF. This is not synonymous with “diagnosis.” Therefore, users should be aware that the program should only be used as an auxiliary tool for medical treatment. As clearly stated in the package insert,^31^ “medical professionals and users must not diagnose arrhythmia or take medical action, including treatment, based solely on the judgment made using the application.”

#### 2.6.2 AF History Program

The AF history program identifies the time of episodes that suggest AF based on analysis of pulse data, and notifies the user the time as a percentage of total time spent wearing the Apple Watch®.^32^ It should be noted that this program can only be used by patients with confirmed AF. Because the program generates and displays estimates every 7 days, it can be used as a reference for assessing so‐called “AF burden,” but as noted above, this is not synonymous with “diagnosis.”

PPG can be a useful tool because it detects AF in real time and continuously, but no devices with an AF detection function have received regulatory approval as yet. For this reason, in patients who test positive in screening, a definitive diagnosis of AF can only be made if confirmed by a physician in a 1‐lead ECG recording of ≥30 s or more or in a 12‐lead ECG.^12^ In order for PPG to become more practical, continuous efforts are needed, such as the development of signal processing methods to remove motion artifacts, the development of interpretable machine learning models, and automation of data annotation.^33^

### 2.7 Heart Rate Monitoring Using the Oscillometric Method

Home‐use digital automatic blood pressure monitors use an oscillometric method to detect irregular pulse waves. The HEM‐907®, made by OMRON, has a sensitivity of 95.5% and a specificity of 96.5% for detecting AF;^34^ the BP785N®, also made by OMRON, has a sensitivity of 100% and a specificity of 84.8% for detecting AF;^35^ the TM‐2441®, made by A&D, has 100% sensitivity and 79% specificity for detecting AF;^36^ and the WS‐X10J®, made by NIHON SEIMITSU SOKKI, has been reported to have 100% (light on/light flashing) and 88.3% (light flashing) sensitivity and 74.8% (light on/light flashing) and 94.6% (light flashing) specificity for detecting AF.^37^ The pooled estimates of sensitivity and specificity in a meta‐analysis comparing the accuracy of the WatchBP® Home A (Microlife; not yet released in Japan), which has an AF detection algorithm (AF Detector) function, with that of a regular ECG were 95% and 94%, respectively.^38^

The irregular pulse wave detection function using the oscillometric method has not been approved or certified by the Japanese Pharmaceutical Affairs Law. Therefore, it cannot be used for diagnosis in the same way as PPG. However, AF can only be diagnosed if an ECG recording of ≥30 s or more is made for 1 lead, or if confirmed by a doctor using a 12‐lead ECG.^35^ In the case of AF, the pulse interval and pulse strength change with each beat, so the correlation between pulse rate and heart rate, which are calculated from the average pulse interval, is weak when limited to tachycardiac AF. This is because the variability of pulse intensity increases during tachycardiac AF and, as a result, the pulse cannot be detected. Therefore, it should be noted that the ability to detect AF may be reduced in tachycardiac AF.

Table **4** provides an overview, indications, and insurance coverage of the ambulatory and wearable ECG devices discussed in this chapter. It is important to understand the advantages and limitations of each device and to use them appropriately.

**TABLE 4:** Summary of the Functions and Features of Ambulatory and Wearable Electrocardiogram Devices

	Overview	Advantages	Limitations	Indications	Insurance reimbursement
Lead‐based ambulatory ECG devices	Classic, standard Holter ECG Attach the electrode with integrated lead wires to the chest and connect to the main unit 12‐lead ECGs can also be recorded	It is possible to analyze delayed potentials (LPs) and repolarization indices (TWA/TWV) Using a 12‐lead ECG, it is also possible to measure the QT interval and related indices It is also possible to detect Brugada‐type ECG waveforms and evaluate ischemic ST‐T	Although simplified, it is still quite large and requires an analysis device	Can be used to definitively diagnose all types of arrhythmia, including AF	Reimbursed by insurance
Patch ECG devices	The ECG is recorded for long periods of time by attaching the device directly to the skin The electrodes are built in	Continuous recording for long periods of time (up to 14 days) is possible Well tolerated by patients	Only 1 lead can be recorded There are some skin problems, although they are rare	Detailed examination of the cause of palpitations or fainting in patients Screening for AF	Reimbursed by insurance
Garment ECG devices	Special clothing with built‐in electrodes used in combination with an ECG machine	When bathing, it is possible to take the special clothing with built‐in electrodes off, just like normal clothing, and there are fewer skin problems	It is not possible to record an ECG when the clothing is removed	Detailed examination of the cause of palpitations or fainting in patients Screening for AF	Reimbursed by insurance
Handheld ECG devices	When necessary, the patient presses the electrodes built into the device against their hands or chest to record an ECG	An ECG can be recorded easily anytime, anywhere The device can also be used during consultations	Only intermittent, short‐term ECGs can be recorded	Detailed examination of the cause of palpitations Screening for AF	^a^In the case of an outpatient, 150 points can be claimed if the device is used under the guidance of a doctor
Handheld‐based ECG devices using a smartphone	Uses a smartphone to record and store an ECG The fingers of both hands are placed on the ends of the ECG device to record an ECG	An ECG can be recorded at home By closely attaching it to the leg, a pseudo‐6‐lead ECG waveform can be displayed	Only intermittent, short‐term ECGs can be recorded	Detailed examination of the cause of palpitations Screening for AF	^a^In the case of an outpatient, 150 points can be claimed if the device is used under the guidance of a doctor
Smartwatch ECG devices	Used in conjunction with a smartphone Continuously measures the pulse while worn on the wrist By placing a finger on the crown of the head while seated, an ECG‐like waveform is recorded	The pulse can be measured simply by wearing the device	Only intermittent, short‐term ECGs can be recorded	Detailed examination of the cause of palpitations Screening for AF	Not covered by insurance
ECG devices based on sphygmomanometers	A device that combines a sphygmomanometer and an ECG ECGs can be recorded while measuring blood pressure by putting on the cuff and touching the electrodes on the main unit with your fingers	Blood pressure measurement and ECG recording can be done at the same time with 1 device	Only intermittent, short‐term ECGs can be recorded	Detailed examination of the cause of palpitations Screening for AF	^a^In the case of an outpatient, 150 points can be claimed if the device is used under the guidance of a doctor
Photoplethysmography	By measuring changes in blood volume, information on pulse waves associated with heartbeats can be obtained This is built in various wearable devices	Pulse waves can be measured simply by wearing the device	ECG cannot be recorded	Screening for AF	Not covered by insurance
Oscillometric method	A function for detecting irregular pulse waves used in combination with home blood pressure monitors	Irregular pulse waves (arrhythmia) can be detected when measuring blood pressure	ECGs cannot be recorded	Screening for AF	Not covered by insurance

^a^ Unit price for 1 point is 10 yen. AF, atrial fibrillation; ECG, electrocardiogram; LP, late potential; TWA, T wave alternans; TWV, T wave variability.

## 3 DATA STORAGE AND SECURITY FOR AMBULATORY AND WEARABLE ECG DEVICES

### 3.1 ECG Waveform Data Storage

#### 3.1.1 ECGs as Digital Data

In the past, ECGs were recorded as analog signals on paper, but in recent years many digital ECG devices have been developed that can store data digitally. Unlike the conventional 12‐lead analog ECG, the digital ECG consists of limb leads (6 leads synthesized from Leads I and II) and chest leads (V1–V6), with each lead consisting of waveform information recorded at a fixed sampling interval (~1–2 ms) and fixed resolution (~0.3–5 *μ*V). This waveform information is time series data, and is a matrix of time×potential height for each lead. A standard 12‐lead ECG recording of approximately 10 s corresponds to approximately 20 kB in data volume for each manufacturer's ECG device, and even after conversion to Medical waveform Format Encoding Rules (MFER; see below) is often around 80–90 kB.

#### 3.1.2 Storing ECG Data

In addition to waveform information, ECG data contains a wide range of other information, including measured values (e.g., PQ time), the results of automatic analyses made by each of the devices, the results of doctors' evaluation, the Minnesota Code, meta‐information (e.g., patients' examination information), and personal information.

There have been many attempts over many years to digitally store electrocardiogram waveforms, but each ECG device manufacturer stored them in unique formats, with no data compatibility between different manufacturers. Therefore, in 2007 the Medical waveform Format Encoding Rules (MFER) were published in Japan, which became an international standard in 2015.^39^

The MFER is a standard that is specialized for medical waveforms. It can store analysed data, meta‐information, and waveforms. It can also describe Holter ECGs and electroencephalograms. Because the specifications are completely open and the viewer is readily available, anyone can restore and analyze the data, making it easy to use in clinical and research settings. Other standard formats for ECG waveforms include SCP‐ECG, US Food and Drug Administration eXtensible Markup Language (FDA XML), and Digital Imaging and Communications in Medicine (DICOM; Table **5**). These standard formats enable data sharing between facilities and manufacturers and have significant advantages in both clinical and research settings, but they are not as widely used as proprietary formats optimized for each manufacturer's system, perhaps because they offer fewer advantages to manufacturers.

**TABLE 5:** Features of Each Data Format

Data format	Binary or XML	Advantages	Disadvantages
Proprietary format of each company	Binary	Data can be easily collected and sorted out	Data from other companies cannot be used
MFER	Waveform section: Binary Attribute information: XML	Can be used not only for 12‐lead ECGs, but also for Holter and EEG	MFER is used in some products for research in Europe, but is mainly used in Japan
SEAMAT	Data stored in XML in SS‐MIX2 format; waveform stored in MFER	It is possible to collect various data, not just ECGs	SEAMAT is used in some products for research in Europe, but is mainly used in Japan
FDA XML	XML	Because it is in XML format, waveform analysis is easy	Japanese manufacturers do not support FDA XML
DICOM waveform	Binary	Because the information obtained is linked to image data, such as ultrasound and CT scans, all these data can be used for research at the same time	There are few Japanese manufacturers that support DICOM

CT, computed tomography; ECG, electrocardiogram; EEG, electroencephalogram; ICOM, Digital Imaging and Communications in Medicine; FDA, US Food and Drug Administration; MFER, Medical waveform Format Encoding Rules; SEAMAT, Standard Export datA forMAT; XML, eXtensible Markup Language.

#### 3.1.3 Data Storage by Ambulatory ECG Devices and Implantable Cardiac Devices

The ECGs obtained with ambulatory ECG devices and implantable cardiac devices are also generally stored in each manufacturer's unique format, exported to PDF, and sent to a hospital's electronic medical records or remote monitoring websites. Although images such as PDF are suitable for visual confirmation by humans, they are not suitable for data utilization. Many of these devices can use Application Programming Interfaces (APIs) to import and store ECG and other numerical data in facilities' databases using standards such as MFER and Health Level Seven (HL7), facilitating data usage.

#### 3.1.4 Storing ECG Data for Research Use

As mentioned above, in many cases ECG data are still stored in each manufacturer's unique format. This is suitable for data use within each company, but because these formats are not compatible, it can sometimes be a problem for use in, for example, multicenter studies. However, by using a manufacturer's proprietary software and storing the data in MFER format, it is possible for the data to be used in multicenter studies.

In 2015, the Japanese Circulation Society created the JCS Data Output Standard Format Guidelines (SEAMAT: Standard Export datA forMAT), which set out the output format for data, including ECGs.^40^ SEAMAT stipulates that the data storage structure based on SSMIX2 should be used to output ECG measurement data and meta information and that MFER and other waveform data should be included in this structure. SEAMAT is likely to be used as a standard for cardiovascular data communication in the future, so it is advisable to consider how to deal with it.

### 3.2 Security of ECG Data

#### 3.2.1 Relationship Between Security and Privacy

Although digital transformation through information technology is progressing, there are issues that need to be resolved in order for it to be used appropriately in medical settings. Clinical activities using information and communication technology (ICT) require measures to be taken for cybersecurity and the protection of personal information as medical information created and stored within or outside medical institutions is shared via information networks.

For example, in the case of ambulatory/wearable ECG devices, Silva‐Trujillo et al. integrated 4 existing reports and reported that there are cases of attacks such as eavesdropping, traffic analysis, information gathering, modification, masquerade, denial of service, replay attacks, attacks based on network properties, malevolent code, and phishing.^41^ Although countermeasures to these sorts of attacks are necessary, it is difficult to implement them perfectly due to factors such as cost and limitations on memory, computing power, and battery life; thus, it has been suggested that organizations and businesses need to design so‐called “defense in depth” (i.e., even if a user leaks their password, the data others can access is limited).^42^ The number of cases of cyber attacks targeting medical information is increasing year by year, and it is essential that measures are taken against the threat of infection of medical information systems by malware, including ransomware, such as WannaCry,^43^ Petya,^44^ LockBit, Emotet, and Elbie.^45^ In addition, users themselves need to be careful about the risk of personal information and privacy being leaked due to the theft of wearable devices.

#### 3.2.2 Guidelines and Guidance on Medical Information System Security

In Japan, medical information systems are based on the so‐called “2G3M” (Two Guidelines from Three Ministries), namely The Safety Management Guidelines for Providers of Information Systems and Services Handling Medical Information^46^ from the Ministry of Internal Affairs and Communications (MIC) and the Ministry of Economy, Trade and Industry (METI) and Guidelines on Safety Management of Healthcare Information Systems^47^ from the Ministry of Health, Labour and Welfare, which consist of guidelines for business operators and medical practitioners. The Research and Development Guidelines for Software as a Medical Device in Medical and Healthcare Fields to Promote Behavior Change^48^ also contain useful information on cybersecurity. When using medical information systems in clinical practice, medical device developers and service providers, as well as the medical professionals who use these devices/services, are required to work together to ensure the safe management and security of patient data.

#### 3.2.3 Protection of Personal Information

Because ECGs are personal information or digitized personal data, it is necessary to establish a system for protecting personal information in accordance with the law when using them. In Japan, the first law concerning the protection of personal information, the *Act on the Protection of Personal Information*, was fully enforced in 2005, after which digitization progressed and utilization accelerated. Furthermore, the *Amended Act on the Protection of Personal Information* was enacted in 2017 and 2022.^49^ In addition, *The Next Generation Medical Infrastructure Act (Act on Anonymized Medical Data That Are Meant to Contribute to Research and Development in the Medical Field)*, a special law in the medical field, was enacted in 2018, and *The Amended Next Generation Medical Infrastructure Act (Act on Anonymized Medical Data and Pseudonymized Medical Data That Are Meant to Contribute to Research and Development in the Medical Field)* was enacted in 2024.^50^ However, there are some areas where the interpretation of these Acts is somewhat fluid, and particular consideration is needed regarding the secondary use of medical information. In addition, when using information in the development of pharmaceuticals and medical devices, as well as when handling patient information in clinical trials, relevant guidance should be followed, such as the Ministerial Ordinance on Good Clinical Practice for Drugs^51^ and the Notice on the Handling of Performance Evaluation Tests for Diagnostic Medical Devices Using Existing Medical Image Data. That Do Not Involve Additional Invasive Procedure or Intervention (PSEHB/MDED Notification No.0929–1).^103^

#### 3.2.4 Practical Management

In the clinical management of ECG recording devices, it is important for medical professionals to have an appropriate understanding of the current situation and issues regarding information security and personal information protection measures. Furthermore, it is necessary to provide patients with appropriate explanations, obtain informed consent, and undertake joint decision making regarding these issues and benefits. In the context of safety management in collaboration with business operators, it is necessary to respond quickly to rapid changes in both regulations and deregulation, as well as technological innovations in security and privacy. In particular, as the number of cases in which ECG data are used in combination with other medical and daily activity data increases, so does the risk of individuals being identified; thus, sufficient consideration of security measures is required. In addition, the Ministry of Health, Labour and Welfare's guidelines^47^ also mention the need to provide patients with information on how to contact the medical institution or another relevant party in the event that there is a problem with the device or it malfunctions, and/or when it is difficult to secure information stored by wearable or home‐based internet of things (IoT) devices are lent to patients.

**FIGURE 4:**
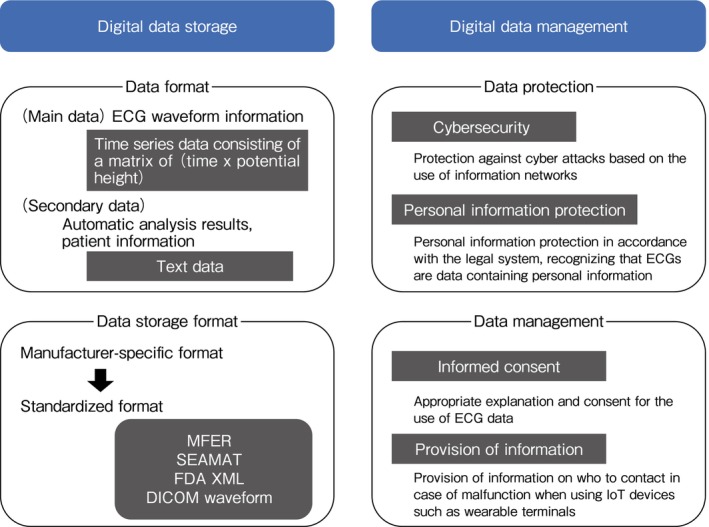
Storing and managing digital data. ECG, electrocardiogram; IoT, internet of things.

Figure **4** shows an overview of the storage and management requirements for digital data obtained from ambulatory/wearable ECG devices described in this chapter.

## 4 HOW TO USE PORTABLE / WEARABLE ECG DEVICES DIFFERENTLY FROM ILRs

### 4.1 Functions and Accuracy of ILRs

#### 4.1.1 What Is an ILR?

An ILR is a device that is implanted under the skin of the anterior chest and continuously monitors the ECG, making it possible to record ECGs during attacks that are difficult to detect even with frequent, long‐term monitoring. In Japan, ILRs are covered by health insurance for 2 purposes, namely to detect unexplained syncope and subclinical AF in patients with latent cerebral infarction.

**FIGURE 5:**
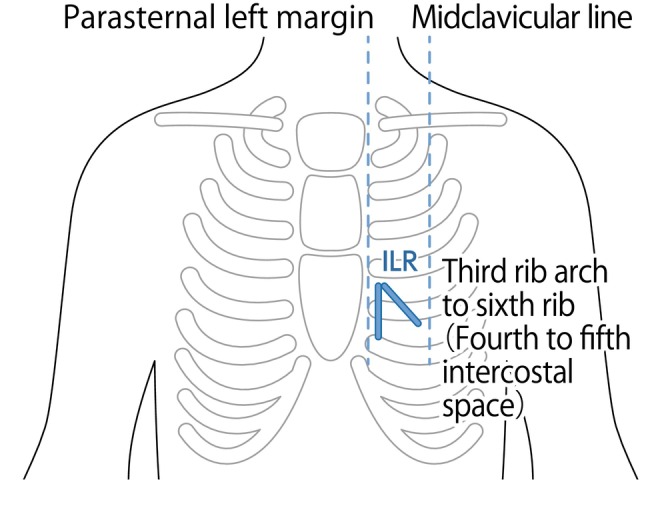
Implantable loop recorder (ILR) placement site.

The ILR device is implanted under local anesthesia using a specialized implantation tool. This is a minimally invasive, simple procedure. Although there are slight differences depending on the manufacturer, as shown in Figure **5**, ILRs are usually implanted from the left parasternal left margin into the third to sixth rib arch (between the fourth and fifth ribs) on the midline of the clavicle.

Even if the patients themselves do not record events, if a preset abnormal heart rate (bradycardia, cardiac arrest, tachycardia, AF) occurs, the ECG is automatically saved. In addition to during regular checkups at hospital, remote monitoring is also possible, enabling early identification of the underlying disease. Complications include postimplantation pain, local bleeding, hematoma, superficial infection, and device migration, but these are rare. After implantation, the patient can record events when symptoms occur, in which case the ECG is saved going back several minutes.

#### 4.1.2 Diagnosis of Syncope of Unknown Cause

The diagnosis of syncope is extremely difficult, and there are reports that even after various tests approximately half of suspected cases could not be diagnosed as syncope.^52^ Syncope is broadly classified as reflex (due to orthostatic hypotension) and cardiogenic, but early diagnosis is important for cardiogenic syncope because the prognosis is poor.^53^ In the Place of Reveal In the Care pathway and Treatment of patients with Unexplained Recurrent Syncope (PICTURE) study, ILR implantation was performed in cases of recurrent syncope that had not been diagnosed despite visits to an average of 3 specialists and 13 types of tests.^54^ At 1 year after implantation, 38% of patients had recurrent syncope; 78% of these patients were diagnosed with an ILR, of which 75% being diagnosed with cardiogenic syncope and treated by either implantation of an artificial pacemaker or an implantable cardioverter defibrillator, or catheter ablation.^54^ In a meta‐analysis of 49 studies of ILR that included 4381 patients with unexplained syncope, the overall diagnostic rate was 43.9% with a median follow‐up period of 365 days. The time to diagnosis ranged from 30 to 600 days (median 134 days), and the rate of arrhythmogenic syncope was 26.5% (ventricular arrhythmia: 2.7%; supraventricular arrhythmia: 4.9%; bradyarrhythmia: 18.2%).^54^

A review of randomized clinical trials comparing ILR with conventional diagnostic testing showed no difference in long‐term mortality between participants in the ILR and standard evaluation groups, but indicated that participants in the ILR group had a higher rate of long‐term (12–20 months) diagnosis than those in the standard evaluation group.^55^

The JCS/JHRS 2022 Guideline on Diagnosis and Risk Assessment of Arrhythmia^12^ states that there is a Class I indication for ILR if: (1) a patient has clinical features that suggest cardiac syncope, but a comprehensive evaluation fails to identify the cause of syncope or select a specific treatment; or (2) a patient does not have any clinical features that suggest cardiogenic syncope, but non‐cardiogenic syncope such as reflex syncope or orthostatic hypotension is unlikely, and the attacks are irregular or syncope of unknown cause recur. Compared with other guidelines, the 2017 American College of Cardiology/American Heart Association/Heart Rhythm Society (ACC/AHA/HRS) guidelines do not include any Class I indications for ILR, and broadly indicate (Class IIa) ILR in cases where arrhythmogenicity is suspected.^56^ Conversely, the 2018 European Society of Cardiology guidelines^57^ state a Class I indication for ILR in patients with: (1) recurrent unexplained syncope, no high‐risk factor, and a high probability of recurrence within the battery life of the device; and (2) high‐risk factors for whom the cause of syncope has not been proven, no treatment has been conducted, and there is no indication for an implantable cardioverter defibrillator or pacemaker. These indications are relatively close to those in the Japanese guidelines. The use of an ILR in patients with suspected or confirmed reflex syncope who present with frequent or severe syncope episodes is classified as a Class IIa indication.^57^

#### 4.1.3 Detection of AF in Patients With Cryptogenic Stroke

The Study of Continuous Cardiac Monitoring to Assess Atrial Fibrillation After Cryptogenic Stroke (CRYSTAL‐AF) was a randomized controlled trial that compared the detection rate of AF using long‐term ILR ECG monitoring with that using standard ECG monitoring as a control in 441 patients with cryptogenic stroke.^58^ The rate of initial AF detection after 6 months was 8.9% in the ILR group, compared with 1.4% in the control group; after 12 months it was 12.4% in the ILR group and 2.0% in the control group.^58^ Among the cases detected, 79% were asymptomatic AF, and it is possible that they would not have been detected using any method other than ILR.^58^ A review of 4 studies (1102 patients) comparing an ILR group with a conventional diagnosis group reported that the AF detection rate was 2.46‐fold higher, anticoagulant therapy was started 2.07 more often, and recurrent cerebral infarction was 0.45‐fold less frequent in the ILR group, suggesting that the use of ILR may contribute to a reduction in the incidence of recurrent cerebral infarction in patients with subclinical cerebral infarction.^59^ It has been reported that the AF detection rate in patients with subclinical cerebral infarction is 20–30%, and many of these cases are asymptomatic.^60^

The Japanese guidelines on the diagnosis and risk assessment of arrhythmia^12^ also recommend (Class I) the use of ILR to detect AF as the cause of silent cerebral infarction when the cause cannot be identified even with long‐term ECG tests, including Holter ECG, in patients diagnosed with silent cerebral infarction.

#### 4.1.4 Currently Available ILR

There are currently 3 types of ILR, and their characteristics are summarized in Table **6**. Compared to the first generation of ILRs, the battery life of these products has increased considerably, ranging from 4.5 to 6.6 years. The Assert‐IQ™ and LINQ II™ have the advantage of being quite compact. The BIOMONITOR IIIm is slightly longer than the others because it has a sufficient distance between electrodes to ensure sensing efficiency.^61^

**TABLE 6:** Features of Implantable Loop Recorders (3 Models)

Manufacturer	Abbott	Medtronic	BIOTRONIK
Product name	Assert‐IQ™	LINQ II™	BIOMONITOR IIIm
Appearance	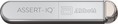	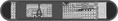	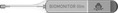
Battery life (years)	6.6	4.5	5.5
Size (capacity/dimensions)	1.9 mL 49.5 × 9.5 × 4.4 mm	1.4 mL 45.1 × 8.0 × 4.2 mm	1.9 mL 77.5 × 8.6 × 4.6 mm
ECG transmission	Selectable between all or 3 ECGs	3 ECGs/10 logs	6 ECGs
Recording	Manual: 4–14 min before/30–60 s after Automatic: 10–60 s (AF 150 s)	Manual: 9–14 min before/1 min after Automatic: 30 s before end, 27 s before end (AF 120 s)	Manual: 7 min before/30 s after Automatic: 30 s before detection, 10 s after detection
Method of recording subjective symptoms (manual recording)	Smartphone app	Dedicated portable device, smartphone app	Dedicated portable device
Remote monitoring	Yes	Yes	Yes
Remote terminal	Smartphone app	Stationary device, smartphone app	Dedicated portable device
Remote setting function	Yes	Yes	No
Other functions	HRV, activity, posture, PVC burden	HRV, activity, PVC burden	HRV, activity, PVC count/day

AF, atrial fibrillation; ECG, electrocardiogram; HRV, heart rate variability; PVC, premature ventricular contraction.

With regard to the important diagnostic functions of these ILRs, all companies have added algorithms to reduce the rate of false positives, and have improved the accuracy of arrhythmia detection. In recent years, algorithms have been added to reduce the false positive detection of pauses or bradycardia when the R‐wave amplitude decreases or there is poor contact between the electrodes and tissue. Algorithms have also been added to reduce the false positive detection of tachycardia in the presence of noise.

The different companies are also strengthening the ability of their devices to detect AF. For example, the LINQ II™ uses Lorentz plots and preceding P waves to examine the variability of RR intervals. By strengthening the ability to differentiate atrial and ventricular premature contractions, and by improving the algorithm to reduce the rate of false‐positive detections, the positive predictive value for atrial tachycardia/AF is now reported to be 95.3%.^104^ The Assert‐IQ™ has a 30‐s predetection window that makes it possible to determine the AF detection interval and waveform, as well as the presence or absence of P waves, and offers a 5‐level AF detection and discrimination function. Compared with previous models, the inappropriate detection rate has been reduced by 45.8% in Assert‐IQ™, and it has 97% relative sensitivity.^105^ The BIOMONITOR IIIm is equipped with a function that excludes extrasystoles from irregular RR intervals.

It is also possible to identify ventricular extrasystoles. Both the Assert‐IQ™ and LINQ II™ use R‐R interval patterns and waveform analysis, and it is possible to evaluate the premature ventricular contraction burden with each model. It has been reported that the LINQ II™ ventricular extrasystole detection algorithm has a specificity of 99.6%.^62^ The Assert‐IQ™ can also detect double and triple ventricular extrasystoles, and is equipped with a ventricular extrasystole detection algorithm that has a specificity of 99.7%.^106^ Other models are also equipped with functions such as a display of heart rate and activity variations, and changes in body position when an arrhythmia is detected.

The length of time for recording ECGs during events is about the same for all models, but the method for recording subjective symptoms (manual recording) differs between companies. The ASSERT‐IQ™ is only compatible with smartphone apps, whereas the BIOMONITOR IIIm is a dedicated portable device. The LINQ II™ can be used with either an app or as a dedicated portable device. Regarding remote monitoring, although there are differences in support tarminals, all the devices have remote monitoring functions. The Assert‐IQ™ allows transmission of electrograms in all cases. The LINQ II™ and Assert‐IQ™ have the advantage of being able to be programmed remotely.

### 4.2 Differences Between Ambulatory / Wearable ECG Devices (Including External Loop ECG Devices) and ILRs

An ILR is an ECG device that is inserted under the skin of the anterior chest for the purpose of detecting infrequent paroxysmal arrhythmias. It is capable of recording events when symptoms occur, and it stores ECGs when preset abnormal heartbeats (bradycardia, cardiac arrest, tachycardia, AF) occur, and has a battery life of 3–5 years.

An ambulatory handheld ECG device (event ECG) is a device that records an ECG after the event button on the recorder is pushed and the device is pressed to the chest during an attack. These devices are very useful for patients with symptoms. They can be used to determine the causal relationship between symptoms and arrhythmia.^7^ An external loop ECG device records an ECG by using electrodes attached to the chest. This is a loop type device that allows ECG recording to be traced back several tens of seconds to several minutes from the onset of the attack. In addition, the ECG is automatically recorded when a preset abnormal heart rate occurs. External loop ECG devices are also useful for detecting arrhythmia in patients without symptoms. Functionally, the external loop ECG device is almost the same as the ILR, but the maximum wearing period is 2–4 weeks.

An example of a wearable long‐term ECG device is the Holter monitor, which can record for long periods of time. With wearable long‐term ECG devices, it is possible to continuously record an ECG for up to 14 days. However, because these devices can only record on 1 channel and the distance between electrodes is short, the quality of the recording, such as differentiation between supraventricular and ventricular arrhythmias, is inferior to that of a Holter ECG,^63^ but sufficient for differentiating basic waveforms such as pauses and tachycardia.

In Japan, the use of an ILR is reimbursed under health insurance only when it is used to detect AF in patients with unexplained syncope and silent cerebral infarction. Ambulatory handheld ECG devices requires patients to record the ECG themselves, and are therefore more suitable for symptomatic patients. They are not suitable for detecting asymptomatic AF in patients with syncope or silent cerebral infarction, and are not suitable as a substitute for ILRs. In a study of 60 patients with unexplained loss of consciousness, the diagnostic rate after 1 year was significantly (*p* = 0.012) higher in the ILR group (52%) and compared with the conventional method group (external loop ECG devices+.

tilt test+electrophysiological study; 20%).^64^ The use of an ILR is more suitable in patients with unexplained syncope than the use of an external loop ECG device, but the ILR is an invasive test for patients and, in some cases, it may not be possible to obtain consent immediately. For patients who are suitable candidates for ILR, it is possible to first use an external loop ECG device or a long‐term ambulatory ECG device to record for several weeks and, if a diagnosis cannot be made with these devices, to then implant an ILR.^65^ For arrhythmic events that occur more than once every few weeks, an external loop ECG device or long‐term ambulatory ECG device is suitable, whereas an ILR is suitable for patients with arrhythmic events that occur less frequently than that^66^ (Table **7**).

**TABLE 7:** Recording Time and Diagnostic Rate for Each Electrocardiograph Recording Device

Recording time	Diagnostic rate (%)	Palpitations	Syncope	Subclinical cerebral infarction
Handheld ECG devices (event ECG)	30 s	50–60	Inappropriate	Inappropriate
Patch ECG devices	Up to 2 weeks	50–70	5–10	5–10
External loop ECG devices	1–4 weeks	70–85	15–25	10–15
ILR	5–6 years	80–90 (not indicated)	30–50	15–20

ECG, electrocardiogram; ILR, implantable loop recorder.

## 5 DIAGNOSIS AND EVALUATION BY TYPE OF ARRHYTHMIA / CONDITION USING AMBULATORY / WEARABLE ECG DEVICES

### 5.1 Detection of AF and Accuracy of Detection

Patch ECG devices that use adhesive gel electrodes can be worn continuously for up to 7 days, improving the AF diagnosis rate.^67^ If the hitoe™ dry electrodes (which are wearable) are used, recording can be continued for 14 days (2 weeks).^68^, ^69^ In a randomized cross‐over study that examined the AF recurrence rate after catheter ablation using dry textile electrodes vs. 24‐h Holter ECG, although the rate of obtaining ECG recordings was lower with the textile electrodes than 24‐h Holter monitoring (82.4% vs. 100%, respectively) due to the effects of cloth changes and noise, the longer‐term recordings resulted in a 3‐fold increase in the AF detection rate.^70^

Ambulatory handheld ECG devices allow patients to record periodically or when symptoms are present, and there are devices that can record from a single lead to 6 leads. The Omron HeartScan HCG‐801‐E® can record for 30 s in a single lead and has been shown to detect AF with a high sensitivity (99%) and specificity (96%) compared with standard ECGs.^71^ A meta‐analysis of 18 studies that examined AF detection using handheld ECG devices reported that they were equivalent to 24‐h Holter monitoring.^14^ However, in the case of asymptomatic AF, the power of detection decreases, so longer recording times are required.

There are 5 companies that sell handheld ECG devices (FDA and CE approved) that use external devices with smartphones. When the accuracy of these devices in identifying AF was investigated, the sensitivity was 58–85% and the specificity was 75–79%, with no statistically significant difference between models.^72^ However, because the rate of assessment failure with the AF detection algorithm is relatively high, ranging from 17% to 24%, it is necessary for a doctor to make a judgment as to the presence of AF based on the ECG.

In the Fitbit® Heart Study published in 2022, a novel algorithm that examines tachograms was used to detect irregular heartbeats (AF). During monitoring with a patch ECG device, AF on an ECG was identified in 221 of 225 patients in whom irregular heartbeats were detected, with positive predictive rate of 98.2%.^73^

The Apple Watch® measures heart rate using PPG and, when an irregular pulse is detected, its algorithm continuously measures changes in heart rate.^74^ In the Apple Heart Study, 2161 of 419,297 participants (0.52%) received an irregular heartbeat notification over the 117‐day monitoring period.^28^ The positive prediction rate for AF detection on the ECG concordant with an irregular heartbeat notification was 84%, whereas when irregular tachograms were detected the positive prediction rate was 71%.^28^

In a study using the Huawei Watch®, published in 2019, suspected AF was detected in 424 of 912 participants (0.23%).^29^ Of the 262 who were effectively followed up, 227 were diagnosed with AF, with a positive prediction rate of 91.6% for PPG.^29^

Many wearable PPGs use algorithms that do not detect arrhythmia during body movement in order to suppress false positives, and because continuous monitoring is not possible there is a possibility that the occurrence of AF is underestimated. In addition to body movement, other factors that can affect the accuracy of PPGs include obesity, dark skin color, tattoos, poor skin perfusion, hypothermia, wrist movement, poor skin contact, and ambient light.^75^ In addition, there are limitations to the heart rate at which AF can be detected. It has been reported that the algorithms cannot detect bradycardic AF at a heart rate of ≤50 beats/min and that the AF detection rate decreases when the heart rate exceeds 100 beats/min.^28^

**FIGURE 6:**
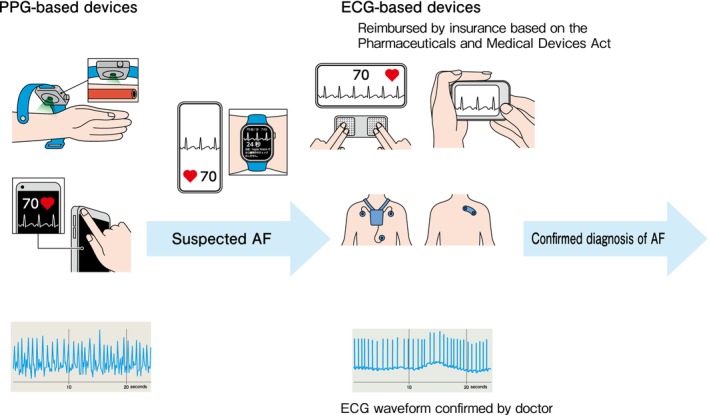
Role of various ambulatory/wearable electrocardiogram (ECG) devices that use photoplethysmography (PPG) and the process up to confirmation of the diagnosis of atrial fibrillation (AF) by a doctor.

The Apple Watch® has been approved as a medical device for the “Home ECG Program [in Japan]”, but the accompanying package insert clearly states that it is not intended for use by people who have been diagnosed with an irregular heartbeat or as a basis for making medical decisions. Therefore, a definitive diagnosis must be made using a long‐term ECG device approved on the basis of the Pharmaceutical Affairs Law or an ambulatory/wearable ECG device that is covered by health insurance (Figure **6**).

A consensus statement on the detection of AF and its accuracy is presented in Table **8**.

**TABLE 8:** Consensus Statement: Detection of Atrial Fibrillation

Detection of AF	Recommendation class
Ambulatory ECG devices, patch and garment ECG devices, and handheld ECG devices in the diagnosis of paroxysmal AF	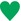
Wearable ECG devices (e.g., smartwatch with photoplethysmography) as an adjunct in suspecting recurrence of paroxysmal AF	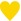
Wearable ECG devices (e.g., smartwatch with photoplethysmography) in the initial diagnosis of paroxysmal AF	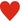
Medication or catheter ablation for AF suspected with a smartwatch‐like wearable ECG device	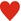

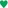
 , recommended for use; 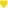 , may be used; 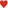 , should not be used; AF, atrial fibrillation; ECG, electrocardiogram.

### 5.2 Evaluation of Arrhythmias Other Than AF

With classic 24‐h Holter monitoring with leads, it is possible to detect almost all tachycardiac and bradycardiac arrhythmias. For bradycardiac arrhythmias, the number and duration of pauses can be quantitatively evaluated. For tachyarrhythmia, the number of supraventricular and ventricular extrasystoles can be quantitatively evaluated using histograms and other methods. In addition, for ventricular extrasystoles, the presence or absence of multiple occurrences, double‐pulse, R on T, ventricular tachycardia. can be displayed, and the frequency of occurrence for each waveform can also be determined. With both the patch and external loop ECG devices, it is possible to quantitatively evaluate the number of ventricular extrasystoles and histograms, but because the analysis is based on a single lead, it may not be sufficiently accurate due to noise, electrode placement, and body position changes, and accuracy is slightly inferior to that of the classic 24‐h Holter monitoring. Handheld ECG devices and ECG devices based on sphygmomanometers cannot quantitatively evaluate arrhythmia. Wearable ECG devices such as smartwatches are not designed to detect arrhythmia other than AF, and cannot be used to diagnose arrhythmia, including AF. It is important to remember that the accuracy of arrhythmia detection with all types of ambulatory/wearable ECG devices depends on the patient's own understanding of the test.

For the diagnosis of arrhythmia with symptoms such as palpitations, the diagnostic rate is higher with patch ECG devices that can record for longer periods than with the classic Holter ECG devices.^76^ In the case of symptoms that occur suddenly, self‐recording by patients is impossible, so it is recommended that a patch ECG device or an external loop ECG device is used. In cases of high‐risk syncope where the frequency of attacks is low, the use of an ILR is indicated.^12^ Conversely, if symptoms are present but are not serious and the attacks are infrequent, the use of a handheld ECG device is recommended.

A consensus statement on the detection and accuracy of arrhythmias other than AF is presented in Table **9**.

**TABLE 9:** Consensus Statement: Detection of Arrhythmias Other Than Atrial Fibrillation

Detection of arrhythmias other than AF	Recommendation class
Classical Holter ECG monitoring for the evaluation of ventricular extrasystole	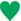
Patch and garment ECG devices or external loop ECG devices for the detection of symptomatic instantaneous arrhythmias	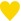

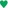
 recommended for use; 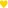 , may be used; AF, atrial fibrillation; ECG, electrocardiogram.

### 5.3 Use in Searching for the Cause of Syncope

If a cardiac cause of syncope is suspected, the involvement of arrhythmia must be considered. In diagnosing arrhythmogenic syncope, the gold standard is to look at the relationship between the syncope and the ECG recording. If no recording of the arrhythmia thought to be the cause of syncope is found, the involvement of arrhythmia can be ruled out. If an arrhythmia that is consistent with the cause of the syncope is recorded, even if the patient is asymptomatic, this can be a basis for diagnosis. For efficient investigation of the cause, it is important to choose the appropriate ECG device according to the frequency of syncope. Monitoring using an ECG device is necessary for high‐risk patients in whom cardiogenic syncope is suspected, and for patients immediately after a syncope. Although there are variations in diagnostic rates between different ECG devices, it is recommended that monitoring using ECG devices be done in order to quickly avoid the risk of another syncope.^57^, ^77^

Because the recording time using classical Holter monitoring is short, syncope does not often recur during the period when the monitor is being worn, and its diagnostic effectiveness is limited.^78^ In cases where syncope or its premonitory symptoms occur frequently, it is useful to clarify whether arrhythmia is involved in the syncope by demonstrating the correlation between the syncope and the ECG. Because handheld ECG devices are not loop‐type devices, they cannot be used for patients who experience paroxysmal syncope. However, they can be used to investigate the cause if symptoms such as palpitations persist after syncope. Smartphone ECG apps cannot be used to investigate the cause of syncope because they cannot accurately detect arrhythmia.

External loop ECG devices are positioned between classical Holter monitor and ILR devices. External loop ECG devices have an automatic trigger function, so can record asymptomatic events, and, because they are a loop‐type device, can obtain ECGs retrospectively by manual operation after the onset of symptoms. In particular, using external loop ECG devices early after syncope improves the diagnostic rate.^6^ These devices are non‐invasive and impose a low economic burden. The ILR is suitable in cases where syncope is rare; like the external loop ECG devices, ILRs can record ECGs during syncope using automatic trigger and loop functions, and their diagnostic rate is higher than that of external loop ECG devices. Although ILRs requires invasive procedures and are expensive, it has been reported that their diagnostic rate is 3.7‐fold higher than that of conventional portable ECG devices, including Holter monitors, and that they are highly cost‐effective.^57^

**FIGURE 7:**
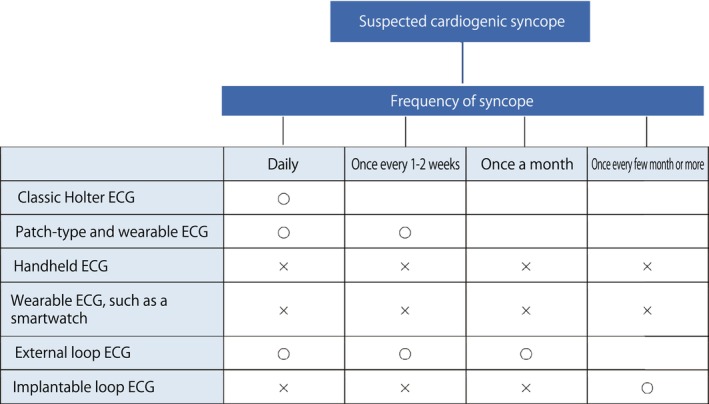
Electrocardiogram (ECG) devices to be used according to the frequency of syncope.

Depending on the frequency of syncope, consider using a classical Holter monitor if syncope occurs at least once a day, a patch or garment ECG device if it occurs once every 1–2 weeks, an external loop ECG devices if it occurs less than once a month, and an ILR if it occurs less than once every few months or years (Figure **7**).

A consensus statement on ECG devices to be used for syncope is presented in Table **10**.

**TABLE 10:** Consensus Statement: Electrocardiographs to Be Used for the Detection of Syncope

ECG devices to be used for the detection of syncope	Recommendation class
Use of classical Holter ECG monitoring, patch and garment ECG devices, and external loop ECG devices in patients with suspected arrhythmogenic syncope	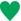
Use of ILR in patients with rare syncope in which arrhythmia is suspected as the cause	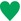
Use of smartwatch ECG apps to find the cause in patients with syncope	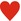

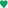
 recommended for use; 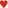 , should not be used; ECG, electrocardiogram; ILR, implantable loop recorder.

### 5.4 Use in Predicting Sudden Cardiac Death

High‐resolution ECGs obtained using Holter monitors can be used to analyze indicators of sudden cardiac death. The following sections describe the indicators of sudden cardiac death that have been shown to be useful in Japan.

#### 5.4.1 Ventricular Late Potentials

Ventricular late potentials (LPs) are indicators of abnormal myocardial depolarization.^79^, ^80^ It is now possible to measure LPs using high‐resolution ECGs obtained using Holter monitors, and, when combined with heart rate turbulence and non‐sustained ventricular tachycardia (NSVT; see below),^81^, ^82^, ^83^, ^84^, ^85^ the measurement of LPs is useful for risk stratification, particularly in post‐myocardial infarction patients who have preserved left ventricular ejection fraction. In arrhythmogenic (right ventricular) cardiomyopathy, ventricular LPs are included as a subitem in the diagnostic criteria.^86^ In the case of Brugada syndrome, ventricular LPs have also been reported to be useful for predicting fatal arrhythmia.^87^, ^88^

#### 5.4.2 T Wave Alternans

T wave alternans (TWA) is a phenomenon in which T waves of different shapes appear alternately at each heartbeat, reflecting abnormal repolarization.^89^, ^90^ TWA recorded using the spectral method (frequency domain method) during exercise stress testing is useful for assessing the risk of fatal arrhythmia and sudden death, mainly in patients with ischemic heart disease.^91^, ^92^, ^93^, ^94^ TWA recorded using the Modified Moving Average (MMA) method (time domain method) with a high‐resolution monitor^90^ is more useful when combined with other non‐invasive indices, such as LPs and NSVTs.^95^

#### 5.4.3 Heart Rate Variability

HRV quantifies the variability of RR intervals and evaluates autonomic nervous system function. The most common methods for evaluating HRV are time domain analysis, represented by the standard deviation of the NN interval (SDNN), and spectral analysis, represented by low frequency and high frequency.^96^ There is greater evidence for the use of SDNN and, in particular, it is associated with sudden death after myocardial infarction.^97^

#### 5.4.4 Heart Rate Turbulence

Heart rate turbulence is an indicator that reflects a decrease in parasympathetic nervous system function, based on the heart rate response to transient blood pressure drops after ventricular extrasystoles, which is mediated by vagal nerve stimulation via the carotid baroreceptor reflex.^98^ When combined with TWA, heart rate turbulence becomes more useful.^99^, ^100^, ^101^, ^102^ The Japanese Multicenter Observational Prospective Study (JANIES) study showed that the combination of heart rate turbulence and NSVT increased the accuracy of prediction of cardiac death.^81^

Table **11** lists the devices currently available in Japan that can measure the 4 predictors of cardiac sudden death listed above, as well as the predictors that each of the devices can measure. A consensus statement on the use of predictive indicators is presented in Table **12**.

**TABLE 11:** Holter Monitors and Analytical Devices That Can Be Used in Japan and the Indicators of Sudden Cardiac Death They Record

	LPs	TWA	HRT	HRV
**FUKUDA DENSHI** Holter recorder Digital Walk FM‐1500 (terminal) Holter ECG analysis system SCM‐9000 (analytical device)	○	○	○	○
**NIHON KOHDEN** Long‐term ECG recorder Cardiomemory RAC‐5203 (terminal) Long‐term ECG analysis device DSC‐5000 series (analytical device)	○	○	○	○
**GE HealthCare** SEER1000 Holter recorder (terminal) SEER12 Holter recorder Holter analysis workstation CardioDay (analytical device)	NA	○	○	○
**MicroPort®CRM** SpiderView® LA456 (terminal) SyneScope® (analytical software)	○	NA	NA	○

HRT, heart rate turbulence; HRV, heart rate variability; LPs, late potentials; NA, not available; TWA, T wave alternans.

**TABLE 12:** **Consensus Statement: Use of Predictive Indicators**.

Use of Predictive indicators	Recommendation class
Evaluation of LP in the diagnosis of arrhythmogenic (right ventricular) cardiomyopathy	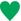
Evaluation of LPs, TWA, or HRT in combination with NSVT to predict sudden cardiac death	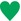
Evaluation of LPs in patients with suspected Brugada syndrome	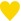
Use of ventricular LPs, TWA, HRV, or HRT alone in risk assessment in patients with post‐MI or non‐ischemic cardiomyopathy	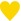

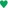
 recommended for use; 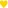 , may be used; HRT, heart rate turbulence; HRV, heart rate variability; LPs, late potentials; MI, myocardial infarction; NSVT, non‐sustained ventricular tachycardia; TWA, T wave alternans.

### 5.5 Application in Athletes

Sudden deaths during and immediately after exercise are often reported in athletes who play competitive sports. For this reason, in addition to monitoring athletes' ECGs in their daily lives, there are cases where athletes need to have their arrhythmia evaluated during competition. In addition, athletes may be monitored during competition to record ECGs during exercise for the purpose of evaluating exercise intensity and assessing cardiopulmonary endurance. There is no difference between the evaluation of ECGs in daily life and normal testing.

Conversely, when recording an ECG during exercise, the conditions are very different from those during normal ECG recording due to problems such as the large amount of noise caused by body movement during exercise, fluctuations in the contact between the electrodes and the skin, and an increase in moisture content due to perspiration; thus, the choice of electrodes is particularly important.

#### 5.5.1 Electrocardiograph

When recording an ECG during exercise, a small ambulatory ECG device that does not interfere with the exercise itself is often used. However, the use of external loop ECG devices is rare, because the event button is difficult to operate and noise contamination can trigger the false recording event. In addition, electrodes are an important element in the recording of an ECG during exercise, and the performance of patch ECG devices with integrated electrodes during exercise needs to be evaluated before their use.

#### 5.5.2 Electrodes

In addition to the discomfort caused by wearing electrodes, electrodes falling off due to perspiration and noise contamination caused by exercise are also problems at athletic‐level exercise. The choice of electrode is particularly important when recording for long periods of time.

Single‐use electrodes that use adhesive substrates or conductive adhesive gel have excellent short‐term adhesiveness, but are poorly waterproof and can easily fall off due to perspiration during exercise. Table **13** shows the characteristics of the substrates involved in electrode crimping. In the case of non‐adhesive conductive gel, rubber, and fiber electrodes, it is necessary to maintain a certain degree of crimping using bands or compression‐type shirts. Perspiration can cause conductive gels to swell, so these gels are not suitable for long‐term monitoring. If you can to maintain a certain degree of crimping using bands or compression‐type shirts, conductive rubber and conductive fibers are excellent in terms of water and sweat resistance, and can be used for long‐term ECG recording.^69^ In addition, many of these electrodes can be reused after washing with water, and can be sewn into a shirt. These shirt‐ and band‐type electrodes are used by placing a small ECG device on the front of the chest.

**TABLE 13:** Features of Substrates Involved in Electrode Crimping

	Solid gel / paste + adhesive substrate‐type electrode	Conductive adhesive gel‐type electrode	Conductive gel	Conductive rubber	Conductive fiber
Adhesiveness	★★★	★★	None^	None^	None^
Water‐ / sweat‐proof	★	★	★★	★★★	★★★
Durability	★	★★	★★	★★★	★★★
Reuse	Not possible	Not possible	★	★★★	★★★

^Requires pressure application using a band or compression‐type shirt. ★★★, very high; ★★, high; ★, within an acceptable range.

## 6 FUTURE PROSPECTS

Ambulatory and wearable ECGs for the detection of arrhythmic episodes are undergoing revolutionary advances, such as miniaturization, wireless connectivity, smartphone integration, longer battery life, and the development of data analysis algorithms. However, manufacturers need to ensure the protection of users' personal information and data security, and work to improve the safety, accuracy, and reliability of the recording devices. Finally, doctors and other medical professionals need to encourage users to use the recording device (ECG) properly. In other words, users need to be aware that the recording device is a “controlled medical device” and “medical device requiring special maintenance,” and that the analysis program is also a “controlled medical device.” Doctors and other medical professionals should not mislead users into thinking that they can use these devices at their own discretion for medical purposes, such as diagnosing, treating, or preventing diseases. That is, it is important that doctors and other medical professionals convey the following 3 points to users:

1. To use these devices, patients must receive instructions from a doctor or medical professional, and be given explanations as to how to use them.
2. Patients should not make self‐diagnosis based on measurement results without the guidance of a doctor.
3. Patients should report both measurement results and subjective symptoms to a doctor.

In terms of future prospects, we can expect improvements in the convenience and accessibility of ECG devices, as well as improvements in data analysis capabilities using AI, applications in telemedicine, and integration with wearable technology. The proper use of ambulatory/wearable ECG devices is expected to contribute to the early detection of heart disease and personalized treatment.

## DISCLOSURES

The COI disclosures in preparing this statement are as follows.

- T.A. received a research grants from NIHON KOHDEN Corporation, and honorarium for lectures from GE HealthCare Corporation, OMRON HEALTHCARE Co., Ltd. and serves on the advisory board of GE HealthCare Corporation, OMRON HEALTHCARE Co., Ltd.
- K.K. received a research grants from JSR Corporation.
- K. Saku received a research funding from OMRON HEALTHCARE Co., Ltd.
- T.S. received a research grants from FUKUDA DENSHI Co., Ltd.
- K. Senoo received a research funding from OMRON HEALTHCARE Co., Ltd.
- S.T. received honorarium for lectures from Medtronic Japan, Abbott Japan LLC, Japan Lifeline Co., Ltd. and is affiliated with the endowed research course supported by Medtronic Japan, Japan Lifeline Co., Ltd., Abbott Japan LLC, and Biotronik Japan.
- M.T. conducts collaborative research with Abbott Japan LLC.
- K.F. has received contributions to an endowed chair from FUKUDA DENSHI Co., Ltd., Medtronic Japan, Biotronik Japan, and SIMPLEX QUANTUM. Research funding has also been provided by Japan Lifeline Co., Ltd.
- W.S. has received remuneration for lectures from Japan Lifeline Co., Ltd., KENZMEDICO Co., Ltd., and OMRON HEALTHCARE Co., Ltd.

## IRB INFORMATION

None.

## APPENDIX. DETAILS OF MEMBERS A

### A.1 WRITING COMMITTEE MEMBERS

- (Group Leader) Takanori Ikeda, MD, Department of Cardiovascular Medicine, Toho University Faculty of Medicine
- Takashi Ashihara, MD, Department of Medical Informatics and Biomedical Engineering, Shiga University of Medical Science
- Yu‐ki Iwasaki, MD, Department of Cardiovascular Medicine, Graduate School of Medicine, Nippon Medical School
- Maki Ono, MD, Department of Cardiology, Kameda Medical Center
- Nobuyuki Kagiyama, MD, Department of Cardiovascular Biology and Medicine, Juntendo University Graduate School of Medicine
- Takehiro Kimura, MD, Department of Cardiology, Keio University School of Medicine
- Kengo Kusano, MD, Department of Cardiovascular Medicine, National Cerebral and Cardiovascular Center
- Ritsuko Kohno, MD, Department of Heart Rhythm Management, University of Occupational and Environmental Health
- Keita Saku, MD, Department of Cardiovascular Dynamics, National Cerebral and Cardiovascular Center
- Tetsuo Sasano, MD, Department of Cardiovascular Medicine, Institute of Science Tokyo
- Keitaro Senoo, MD, Department of Cardiac Arrhythmia Research and Innovation, Kyoto Prefectural University of Medicine
- Seiji Takatsuki, MD, Department of Cardiology, Keio University School of Medicine
- Naohiko Takahashi, MD, Department of Cardiology and Clinical Examination, Faculty of Medicine, Oita University
- Mitsuru Takami, MD, Division of Cardiovascular Medicine, Kobe University Graduate School of Medicine
- Yukiko Nakano, MD, Department of Cardiovascular Medicine, Hiroshima University Graduate School of Biomedical and Health Sciences
- Kenichi Hashimoto, MD, Department of General Medicine, National Defense Medical College
- Katsuhito Fujiu, MD, Department of Cardiovascular Medicine, Graduate School of Medicine, The University of Tokyo
- Tadashi Fujino, MD, Department of Cardiovascular Medicine, Toho University Faculty of Medicine
- Atsushi Mizuno, MD, Department of Cardiovascular Medicine, St. Luke's International Hospital
- Koichiro Yoshioka, MD, Department of Cardiology, Tokai University School of Medicine
- Eiichi Watanabe, MD, Division of Cardiology, Fujita Health University Bantane Hospital

### A.2 EXTERNAL EVALUATION COMMITTEE MEMBERS

- Wataru Shimizu, MD, Department of Cardiovascular Medicine, Graduate School of Medicine, Nippon Medical School
- Koichi Node, MD, Department of Cardiovascular Medicine, Saga University
